# Metabolic STAMP for deciphering GPCR-regulated insulin secretion by pancreatic β cells

**DOI:** 10.1101/2025.10.03.680349

**Published:** 2025-10-04

**Authors:** Mohammad Ovais Aziz-Zanjani, Rachel E. Turn, Yan Hang, Anushweta Asthana, Leilani Elizabeth LaBrie, Mohammadamin Mobedi, Lucy Artemis Xu, Michael Krawitzky, Seung K. Kim, Peter K. Jackson

**Affiliations:** 1 Baxter Laboratory, Department of Microbiology & Immunology, Stanford University School of Medicine, Stanford, CA 94305, USA; 2 Stanford Diabetes Research Center, Department of Developmental Biology, Stanford University School of Medicine, Stanford CA 94305, USA; 3 Bruker Scientific, San Jose, CA, USA; 4 Co-first authors who contributed equally to this manuscript; 5 Co-corresponding authors

**Keywords:** GSIS (glucose-stimulated insulin secretion), GPCRs (G protein-coupled receptors), PTMs (post-translational modifications), MIN6 (mouse β-cell line), primary human islets, Metabolic-STAMP (Synchronized Temporal-Spatial Analysis via Microscopy and Phosphoproteomics), T2D (Type 2 Diabetes), FFAR4 (Free Fatty Acid Receptor 4), GLP1-R (Glucagon-Like Peptide-1 Receptor), ATAT1, HDAC6 (Histone De-Acetylase 6), ERK, PKA (Protein Kinase A), microtubules, acetylation, inter-organellar contacts

## Abstract

Pancreatic β cells integrate glucose and metabolic cues to regulate insulin secretion, a process disrupted in T2D. GPCRs play a critical role in fine-tuning insulin release, yet the mechanisms by which ciliary (*e.g.,* FFAR4) and non-ciliary (*e.g*., GLP1-R) GPCRs coordinate GSIS remains unclear. In this study, we employed Metabolic-STAMP (Synchronized Temporal-Spatial Analysis via Microscopy and Phosphoproteomics) in both mouse β cells (MIN6) and primary human islets to map the dynamic signaling networks governing GSIS and to link transient phosphorylation events to their functional outcomes. We systematically interrogated GPCR-mediated phosphorylation events through selective pharmacological inhibitors, resolving signaling hierarchies and consensus patterns across multiple pathways. Our multi-modal approach uncovered key insulin-secretion-associated PTMs, linked phosphorylation targets with phenotypic organelle dynamics, and provided mechanistic insights into how GLP1-R versus FFAR4 modulates GSIS through shared and GPCR-specific phospho-signatures. We highlighted key examples of stimulus-specific regulation by high glucose alone versus GPCR stimulation, including context-specific activation of the classic ERK signaling pathway, compartmentalized PKA signaling, pathway specificity in organelle dynamics and inter-organellar contacts, and HDAC6/ATAT-mediated regulation of microtubule acetylation. Collectively, these findings provided a blueprint for deconvolving pathway specificity of β cell GPCR signaling, illuminated regulatory nodes that program insulin release, and offered new therapeutic targets to enhance β-cell function.

## INTRODUCTION:

A defining feature of healthy pancreatic β cells is their ability to swiftly and dynamically respond to rapid changes in glucose levels. This heightened sensitivity is largely orchestrated by post-translational modifications (PTMs), notably phosphorylations, which act as rapid and reversible regulatory mechanisms. The transient nature of phosphorylation, controlled by kinases and phosphatases, enables the rapid acceleration of insulin secretion (within minutes) in response to glucose, as well as its prompt cessation when glucose levels drop. Previous groups^1–5^ have mapped the phosphoproteomic responses to glucose stimulated insulin secretion in β cells, yet the cellular functions of these individual modifications remain poorly understood.

Once triggered by glucose, β cell insulin secretion is further modulated by G protein-coupled receptors (GPCRs), which integrate signals from circulating factors including free fatty acids, amino acids, neurotransmitters, and incretin hormones. Among these, FFAR4 (Free Fatty Acid Receptors 4) and GLP1-R (Glucagon-Like Peptide-1 Receptor) have been identified as strong regulators of glucose-stimulated insulin secretion (GSIS), making them important pharmacological targets.^6–9^ However, the mechanisms by which diverse receptors differentially signal to coordinate GSIS remain largely unexplored.

Recent research highlights primary cilia as critical signaling hubs that organize GPCR activity in both α and β cells. Notably, ciliopathies have been linked to impaired insulin secretion and an increased risk of diabetes, emphasizing the importance of ciliary signaling in metabolic regulation. Recent mouse knockouts of primary cilia show that primary cilia are critical for insulin secretion.^10^ Our recent analysis identifies multiple ciliary GPCRs that signal through primary cilia including the omega-3 fatty acid receptor FFAR4.^6^ Stimulation of numerous β cell GPCRs enhance GSIS. Despite extensive studies on these individual GPCRs to understand mechanism, how multiple GPCRs activate similar or complementary signaling pathways to enhance insulin secretion remains unclear. A key unanswered question is how multiple GPCRs modulate kinase activities and downstream phosphorylation events to coordinate β-cell responses to metabolic cues. These phosphorylations have the potential to regulate diverse, context-specific features of protein functionality, including but not limited to negatively or positively regulating enzymatic activity, localization, binding partner affinity, and structural conformation.^11^ The majority of phosphorylation sites identified via bulk phosphoproteomics have no known function to date, and the context-specificity of these modifications remains even more poorly understood.^12^

To address this gap, we employed Metabolic-STAMP to systematically map GPCR-activated kinases and their linked substrates.^13^ Given recent advances in defining consensus phosphorylation patterns (CPPs) linked to specific kinases,^14^ we can define the kinase-CPP pairings that are activated during GSIS. Additionally, every phosphorylation is connected to a specific “timestamp,” reflecting a distinctive functional timed event that is linked to a cellular phenotype defined by microscopy of key cellular markers.^13^ By integrating advanced microscopy connected to these timestamps, we can visualize the dynamics of critical cellular processes—such as microtubule shortening, cilia lengthening, altered mitochondrial morphology, vesicular trafficking dynamics— and kinetically link them to CPP-defined phosphorylations that regulate β cell function. This approach reveals physical changes within the cell and captures the precise localization of proteins whose activity is controlled by phosphorylation, offering a more comprehensive spatiotemporal understanding of insulin secretion. We can correlate these dynamic cellular events with PTMs through metabolic-STAMP, providing a detailed view of how β cells respond rapidly to glucose fluctuations and maintain their functional integrity. This study provides a systems-level blueprint of specific GPCR signaling networks governing GSIS, offering novel insights into the regulatory nodes that control insulin secretion. Moreover, phosphorylations can serve as vital markers for assessing β cell functionality, which is essential for monitoring therapeutic interventions and evaluating the viability of engineered β cells for clinical applications.

## RESULTS:

### Quantitative phosphoproteomics maps stimuli-specific subcellular signaling dynamics in β cells

Despite the identification of many key effectors of insulin secretion, the cell biological and temporal-spatial mechanisms involved in GPCR regulation of insulin secretion remain poorly defined.^15,16^ To address this knowledge gap, we applied the recently developed STAMP workflow^13^ to the glucose-responsive murine β cell line MIN6 to elucidate the GPCR-kinase-substrate signaling network governing insulin secretion.

**Figure 1:**
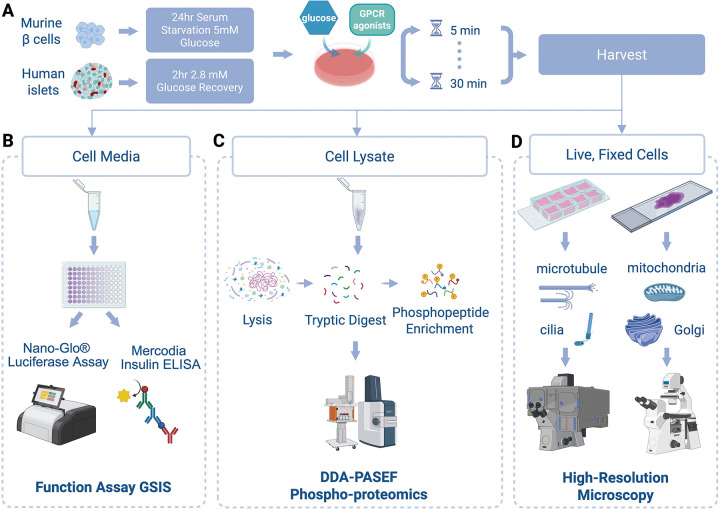
Overview of metabolic STAMP (Synchronized Temporal-Spatial Analysis via Microscopy and Phosphoproteomics) to decipher GPCR-kinase signaling network governing insulin secretion. (**A**) Murine β cells or human islet cells were treated by high glucose alone or together with GPCR agonists to stimulate insulin secretion. During secretion, the following time-controlled samples were collected for metabolic STAMP: cell media, cell lysates, live cells, and fixed cells. (**B**) Cell medium was analyzed for hormone secretion levels like ELISA and/or bioluminescence based high-throughput assays. (**C**) Cell lysates were processed for DDA-PASEF phosphoproteomics to map phosphorylation sites of GPCR signaling pathways. Putative kinases were nominated based on *in silico* analysis and then validated using kinase inhibitor assays. (**D**) Paired live and fixed cell analysis via confocal microscopy were used to assess context specific changes in the following cellular compartments: microtubule cytoskeleton, mitochondria, Golgi and endosomal traffic, and primary cilia.

As a first experiment, we employed DDA-PASEF-based phosphoproteomics to map changes in phosphorylations 10min after cells are exposed to 1) 2.8mM low glucose (LG), 2) 17.8mM high glucose (HG), 3) high glucose with 20nM GLP1-R agonist Exendin4 (Ex4), 4) high glucose with 100μM FFAR4 agonist TUG891 (TUG891), or 5) high glucose with both agonists combined (Combo). This time point was selected based off previous studies^1^ showing maximal changes in phosphosignatures upon glucose-stimulated insulin secretion. From the same cells, we assessed endpoint insulin secretion levels and surveyed subcellular organelles by confocal microscopy in parallel at 5 and 20min (Fig. 1) so that we could assess phenotypes before and after the phosphoproteomic timepoint. Together, these multiplexed approaches provided a platform for linking mechanistic outputs to functional readouts/cellular phenotypes.

**Figure 2:**
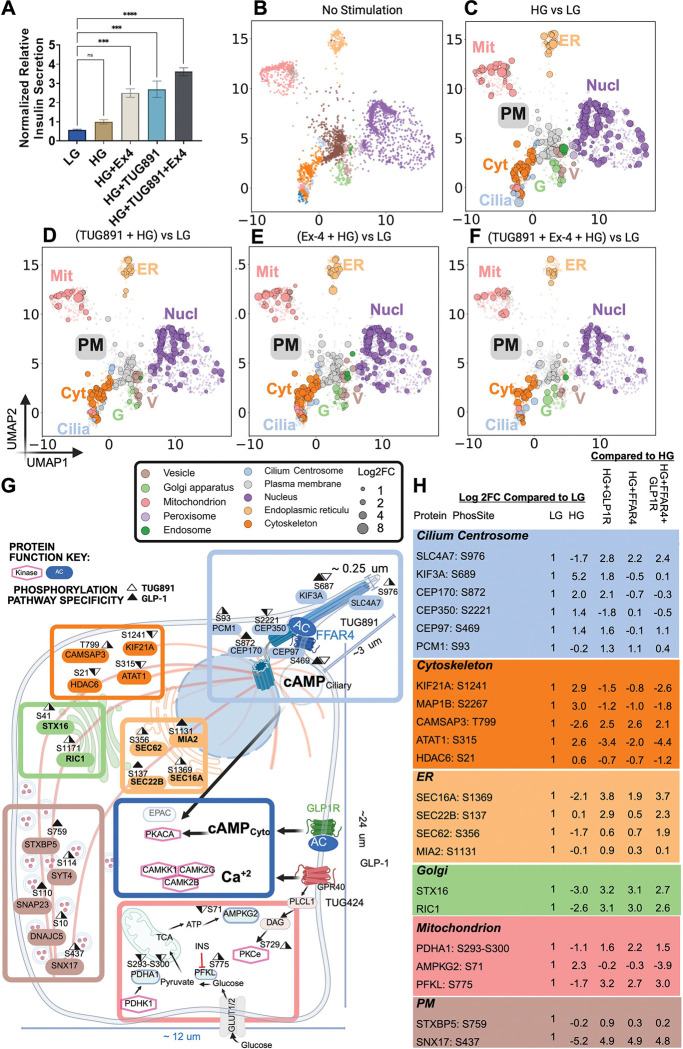
Mapping the differential phosphoproteomics of GSIS with and without co-stimulation with GPCR agonists (**A**) Normalized relative insulin secretion levels were determined via luciferase assay was measured in mouse pancreatic β cell line MIN6 either at low glucose (2.8mM) or treatment with combinations of high glucose (17.8mM), TUG891 (100μM), or Exendin4 (20nM). Cells were serum and glucose starved for 1hr before treatment, and samples were collected at 30 min after treatment. N=3. Error bars= SEM. Statistical significance was assessed via One-Way ANOVA, with *P = 0.05; **P = 0.01; ****P = 0.00001. Plots were generated using GraphPad Prism software. (**B-F**) UMAP analysis reveals the differential effects of glucose and GPCR stimulation (FFAR4 vs GLP1-R) upon the cellular phosphoproteomic landscape. Size of circle indicates log2 fold change, color of circle represents the known subcellular compartment where that protein functions. The first panel indicates the baseline of all the possible phosphorylations detected, while the subsequent panels show increased circle sizes for the phosphorylations detected that show marked changes in abundance in that stimulation condition. (**G**) Phosphorylated proteins are known to be important for the integrity and function of multiple subcellular compartments. These include cilia and centriole (blue), microtubule and kinesins (orange), Golgi and ER (green), secretory machinery (yellow). In addition, some proteins were previously associated with regulatory machinery of GPCR signaling (navy) and glucose metabolism (grey). Example phosphorylations are shown here in a model figure of a β cell, with key cellular compartments marked. Arrowheads indicate whether it is an increase or a decrease in abundance and which GPCRs/stimulations induce change (black: GLP1-R; white: FFAR4). (**H**) Examples of changes in abundance of pathway-specific phosphorylations during GSIS. Differences are reported as log2 fold change compared to low glucose conditions, and the putative kinase based on the Cantley algorithm are listed for each phosphorylation.

As previously observed,^6,17^ both Ex4 and TUG891 enhanced insulin secretion from MIN6 cells ~2–2.5-fold relative to high glucose at 30min, and their combination further amplified insulin response compared to either stimulation alone (~3.5 fold) (Fig. 2A). Mouse pancreatic β cells typically release 1% of their total pool of insulin granules (~9,000–15,000 granules^18^) during GSIS. Therefore, such a strong increase in insulin release upon receptor stimulation suggests the likelihood of new regulatory mechanisms coordinating the releasable pool of insulin granules. To capture key signals driving insulin secretion in different stimulation conditions, we performed phosphoproteomics of LG, HG, HG+Ex4, HG+TUG891, and Combo treatments at 10min after stimulation. Phosphoproteomics quantified >17,000 reproducible, high-confidence phosphorylation sites on >3,700 proteins under five combination conditions (Supplementary Table 1). Most phosphosites (14,794 of 17,567) were shared in multiple conditions, although ~10% were unique to glucose treatment, and another ~9% appeared only with GPCR activation. To profile global changes, we quantified stimulus-dependent dynamics of these phosphosites. After 10min HG treatment, 2,569 proteins showed alterations in phosphorylation levels, including 1550 with elevated levels compared to LG (Supplementary Table 1). Similarly, Ex4 alone, TUG891 alone, and Combo treatments each led to increased phosphorylation levels on >1000 proteins relative to LG. Only a modest fraction of HG-enhanced sites overlapped with those induced by GPCR agonists (26.6% Ex4 vs HG, 27.2% TUG891 vs HG, 10.5% Combo vs HG), suggesting a cluster of phosphorylations consistently linked to insulin secretion, but also a considerable number of context-specific phosphorylations specifically linked to GPCR-mediated signaling (Supplementary Table 1). Strikingly, >800 sites, representing 667 proteins, were induced by HG, Ex4, or TUG891 were attenuated in the Combo treatment (Fig. 2F), the majority of which identified phosphorylations have not been explored in β cells before.

To localize these phosphoproteome changes and assess whether there is compartment-based differential regulation, we annotated proteins with established subcellular markers^13^ and visualized their distribution via UMAP analysis (Fig. 2B). This analysis resolved the entire complement of ~3,700 phosphoproteins into 10 subcellular compartments, highlighting broad effects of glucose versus differential GPCR activation on β cell endophenotypes during GSIS. HG alone primarily altered phosphorylation in the cilium, cytoskeleton, mitochondria, Golgi/ER, plasma membrane, and vesicular compartments (Fig. 2B, C). Compared to LG, Ex4 alone and TUG891 alone induced changes in similar compartments (Fig. 2D, E).

Quantitative phosphoproteomics identified numerous GPCR-agonist-induced phosphoproteins, including kinases, phosphatases, microtubule-associated proteins, ciliary proteins, kinesins, mitochondria, secretory pathway proteins, and protein transport factors not previously characterized in β cells (Fig. 2G). Notably, some phosphorylation sites responded differentially to glucose vs GPCR stimulation, both in magnitude and direction (Fig. 2H). For example, several key phosphorylations on microtubule cytoskeleton machinery show strong opposite fluctuations in abundance in GPCR-stimulation conditions compared to high glucose stimulation alone (see the orange panel in Fig. 2H). These included GPCR-reversed phosphorylations including microtubule associated protein 1B (MAP1B) at [S2267] and microtubule growth regulator kinesin (KIF21A) at [S1241], which are in high abundance upon high glucose stimulation alone but are suppressed by Ex4, TUG891, and Combo treatments. Conversely, phosphorylation of Calmodulin-regulated spectrin-associated protein 3 (CAMSAP3) at [T799] is strongly suppressed in HG stimulation alone but is amplified 2-fold in all 3 GPCR stimulation conditions. CAMSAP3 is a critical regulator of minus end, non-centrosomal microtubules to prevent depolymerization and stabilize the minus end of the microtubule.^19^ The function of this site is unknown to date, but perhaps could be linked to the differential regulation of microtubules based on receptor stimulation, described below.

On the other hand, there were contexts in which different GPCR stimulations showed differential effects on phosphorylation patterns. For example, we observed some key phosphorylations of known ciliary/centrosomal regulators that were differentially regulated by TUG891 versus Ex4 treatment: kinesin KIF3A[S687] and centrosomal protein CEP97[S469]. In both cases, TUG891 stimulation suppresses the phosphorylation to levels below low glucose while Ex4 treatment amplifies the signal. Note that the KIF3A[S687] site is a known regulator of KIF3A activity, as previous studies have shown that phosphorylation at this residue promotes binding of cargoes while blocking binding of membranous cargoes in other contexts.^20,21^ Together, these data provide an extensive map to decode complex signaling networks regulating insulin secretion. These data pointed to trends of both synergy and pathway specificity between different glucose and GPCR stimulation conditions, raising the critical question of how these different pathways work in concert to amplify insulin secretion.

### Stimuli-specific compartmentalized signaling in β cells

Kinases are central to GSIS regulation, as shown by prior loss-of-function studies in β cells.^22,23^ However, little is known about stimuli-responsive phosphorylation signaling pathways, compartmentalization of kinase activity, functionality of these sites, or their effects on downstream substrates in β cells. To this end, we extracted phosphorylation sites across the pathway nodes from quantitative phosphoproteomics data, overlaid them onto a public signaling network (PhosphoPlus^®24^), based on literature-derived observations, and tracked differential activity triggered by glucose versus GPCR stimulation. This targeted approach, which directly tracked the phosphorylation patterns at predefined sites, revealed new insights into well-annotated signaling networks, including the Protein Kinase A (PKA) and Extracellular Signal-Regulated Kinase (ERK) pathways.

**Figure 3:**
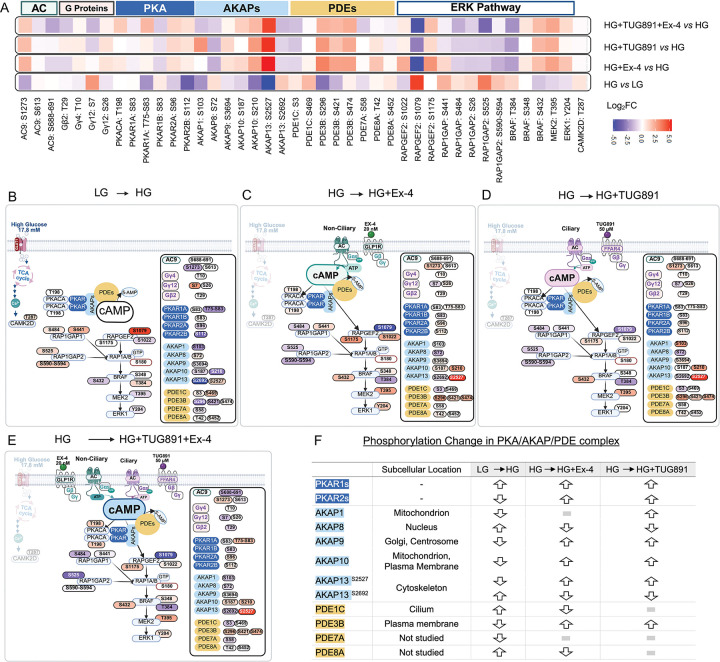
Map of the ERK pathway phosphoproteomic signatures of MIN6 cells in different GSIS regulatory conditions. Here, we highlight the value of our approach for mapping key phosphorylations in known signaling pathways and how they are differentially regulated in the context of GSIS with and without GPCR stimulation. **(A**) Heat map showing the log2 fold change in abundance of ERK pathway (and other key signaling regulators) phosphorylations upon stimulation with high glucose and different GPCR agonists at 15min post-treatment. Phosphorylations are sorted based on protein family, as indicated on the right. Dots are colored as a heat map, with increased abundance being red and decreased abundance being blue. in the schematics on the right, showing (**B-E**) Schematics of the ERK phosphosignaling pathway, indicating which pathways are stimulated under which conditions and how the phospho-signatures vary depending on treatment conditions. Note that high glucose (HG) (**B**), HG+Exendin-4 (**C**), HG+TUG891 (**D**), and HG+TUG891+Exendin-4 combined treatment (**E**) pathway specificity are highlighted in bright color to show which pathways activate which factors. Phosphorylations are shown with heat-map coloration corresponding with the heat-map abundance shown in (**A**). In the box on the bottom left of each panel is a table summarizing the key phosphorylations shown here and the changes in abundances. (**F**) Chart summarizing key major changes in key regulators of the ERK pathway based upon HG versus Exendin4 versus TUG891 stimulations, with up and down arrows indicating either significant increase or decreased levels versus LG for HG or HG for the GPCR stimulations. The second column lists what compartments these different factors localize to, providing spatial context for compartmentalized ERK pathway signaling.

PKA catalytic and regulatory subunits form cAMP-activated signaling complexes, together with the scaffolding protein A-kinase anchoring proteins (AKAPs) and phosphodiesterases (PDEs) that catabolize and inactivate cAMP, orchestrating spatiotemporal regulation of cellular responses.^25,26^ Constitutive phosphorylation of the PKA catalytic subunit at [T198] was abundantly detected across all conditions, validating the robustness of our dataset. Conversely, we observed isoform-specific differential responses among other complex components. For instance, cytoplasmic Type 1 regulatory subunits (*e.g*., PKAR1A[S83], PKAR1B[S83]) responded to glucose, whereas organelle-anchored Type 2 subunits (*e.g*., PKAR2A[S96], PKAR2B[S112]) were induced by GPCR activation (Fig. 3A, B). PKAs were sequestered by distinct AKAP family members in discrete compartments to ensure precise temporal-spatial regulation. Ex4 and TUG891 augmented phosphorylation of AKAP1, AKAP9, and AKAP10 (localized to mitochondria, Golgi, cytoskeleton, and plasma membrane respectively^27,28^), while glucose selectively induced phosphorylation of nuclear AKAPs (Fig. 3A, C). PDEs degrade cAMP, shaping local cAMP gradients to dampen PKA activation.^29^ Glucose induced phosphorylation of cilium-associated PDE1C, whereas GPCR stimulation phosphorylated membrane-bound PDE3B.^30,31^ Notably, FFAR4 activation uniquely elevated phosphorylation of mitochondrial AKAP1 while reducing phosphorylation of mitochondrial PDE8A (Fig. 3A, D), a pattern not observed with either glucose or GLP1-R stimulation. Given the link of FFAR4-dependent rapid activation of ciliary cAMP and ciliary elongation (see below), identifying the key cAMP effectors proteins including PKA regulators like AKAPs and PDEs may reveal strong candidate mediators of these events.

In parallel, we observed stimulus-specific activation of the ERK pathway. GLP1-R activation, but not ciliary FFAR4 activation or glucose, induced a cascade of stimulatory phosphorylations on GAPGEF2, RAP1, BRAF, MEK2, ultimately leading to ERK activation at [Y204] (Fig. 3B–D). These phosphoproteomics findings were corroborated by Western blot (Fig.S1), which confirmed that GLP1-R stimulation enhanced phospho-ERK activity compared to FFAR4. Collectively, these results highlight a mechanism by which distinct GPCR stimuli engage unique signaling modules to control temporally and spatially distinct regulators to organize distinct cytosolic machinery controlling insulin secretion.

### Kinase-inhibitor screen reveals regulators of β cell insulin secretion

**Figure 4:**
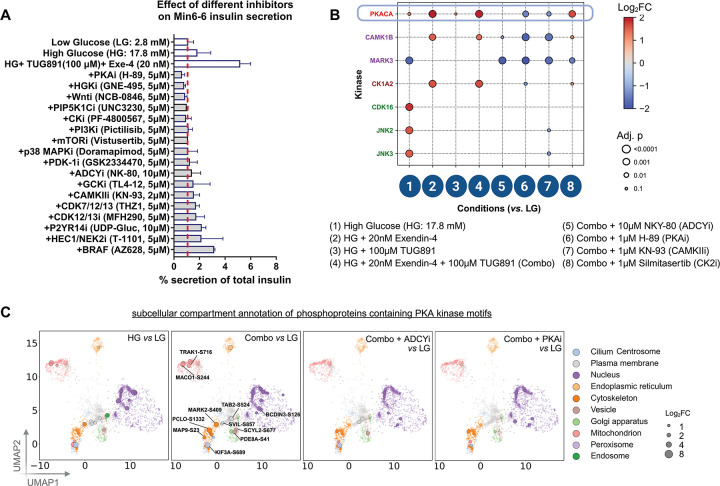
Mapping the context-dependent regulation of kinases during MIN6 GSIS with and without GPCR activation (**A**) Plot of the effects of different inhibitors (ADCY inhibitor, 15 kinase inhibitors, P2YR14 receptor agonist) on % insulin secretion. Error bar= SD. (**B**) Dot plot representing the fold change in abundance of kinase activity. Redder colors indicate an increase in abundance; bluer colors indicate a decrease in abundance. The size of the dot reflects the p value. Each row indicates the activity of a given kinase based on quantitation of phosphorylated consensus sequences (see Figure S3 for workflow), and each column represents a different treatment condition and how those abundances changed. (**C**) UMAPs of compartment localization of known PKA substrates and tracking the differential effect of stimulatory versus inhibitory treatments on their abundance. On the far right, note that the key that indicates which colored circles refer to which compartments, and the size of the circle represents the log2 fold change in phosphorylation abundance.

Our phosphoproteome dataset provided a platform to identify kinases contributing to glucose-stimulated and GPCR-potentiated insulin secretion in β cells. To test functional dependencies, we first screened kinases for their effect on insulin secretion using the Gray kinase inhibitor-focused Library.^32–34^ The library included 240 validated kinase inhibitors, spanning a wide range of protein kinases linked to established core signaling pathways. MIN6 β cells were pretreated for 1hr in low glucose medium with each inhibitor, then stimulated with the combination treatment (HG+ Ex4+ TUG891) in the continued presence of inhibitor. Secreted insulin was quantified 30min later, and inhibitors were categorized by their ability to enhance or suppress GSIS relative to baseline (Fig.S2). In total, at least 17 kinase inhibitors or regulators suppress insulin secretion (Fig. 4A) by 4-fold or more. The screen both validated the importance of kinases with previously known function in insulin secretion (*e.g.* PKA) and additionally identified new kinases that had not been previously associated with insulin secretion regulation (*e.g*. HEC1).

To identify which Combo treatment-induced phosphosites depended on specific kinases, we performed phosphoproteomics on MIN6 β cells treated with glucose, Ex4, and TUG891, together with inhibitors targeting either PKA (H-89), Casein Kinase (Silmitasertib), or CAMK (NK-93). We decided to perform the inhibitor treatments on the Combo, low glucose, and high glucose conditions for comparison to see the maximal effects of loss of kinase activity in the context of GPCR signaling. Kinase motif enrichment analysis (KSEA)^14^ (Methods) confirmed loss of putative substrate phosphorylation under inhibitory conditions (Fig.S3, Supplementary Table 2). For instance, phosphosites enriched in PKA motifs were selectively and strongly reduced after treatment with PKA inhibitor H-89, while sites matching CAMKII motifs were strongly reduced after CAMKII inhibitor KN-93 treatment (Fig. 4B, Supplementary Table 1). While these changes in kinase selective phosphorylations are expected, we also identified novel candidate substrates for PKA (*e.g.*, SEC16A[S1369] and RAB3GAP1[S537]) and for CAMKII (*e.g.*, VAMP2[S75] and SEC22B[S137]). Moreover, the changes in phosphorylations provide validation of *in vivo* CPPs.

What was less expected is that the altered patterns for PKA and CAMKII selective phosphorylations are highly suggestive of crosstalk between these two kinases, as it appears that PKA inhibition was sufficient to suppress some CAMKII motif phosphorylations, and vice versa. Specific substrates, such as MARK2[S409] (a microtubule associated kinase^35,36^), were sensitive to both inhibitors, whereas others were surprisingly uniquely affected by the opposite treatment condition, such as the STXBP5[S693] (a key regulator of calcium-mediated exocytosis^37^) which is a putative CAMKII site but is only suppressed by PKA inhibition. In parallel, inhibition with ADCY inhibitor (NKY-80) revealed overlap with PKA-dependent sites as well as distinctive targets (Fig. 4B, C, Supplementary Table 1). Approximately 50% of the sites that were inhibited with the PKA inhibition were also inhibited with the ADCY inhibition. The lack of complete overlap between the two datasets is to be expected as the 10μM dose that we used was ideal for targeting ADCY5 (the major adenylyl cyclase in β cells), but it has reduced/minimal efficacy for targeting some other adenylyl cyclases including ADCY5.^38^ Future work would be needed to assign which ADCYs are linked to which downstream phosphorylations.

We next asked whether there was compartment-specificity for the mechanisms by which these kinases regulate GSIS. To do so, we also performed subcellular compartment analysis. Our findings revealed that the following subcellular compartments were enriched for putative PKA-phosphorylated proteins: cytoskeleton, cilium, mitochondria, vesicle, and plasma membrane (Fig. 4C). While many of the same sites are suppressed by both ADCY and PKA inhibitor treatments (*e.g.*, cytoskeletal components MAP9[S23] and MARK2[S409] and plasma membrane protein SVIL[S857]), some sites were attenuated by PKAi but were either unaffected or not as strongly suppressed in ADCYi treatment, including ciliary KIF3A[S689] and plasma membrane TAB2[S524]. Together, these kinase-inhibitor studies corroborate our phosphoproteomics results and demonstrate both specificity and convergence of signaling pathways downstream of glucose versus GPCR stimulation in β cells.

### Stimulus-specific remodeling of β cell organelles

To test whether phosphorylation dynamics translate into intracellular remodeling events directing changes in β cell function, we performed high-resolution live and fixed cell microscopy of key compartments that revealed robust, context-specific fluctuations in phospho-signaling: cilia, microtubules, mitochondria, and the Golgi apparatus. We observed examples of both pathway-specificity and synergy for each compartment.

**Figure 5:**
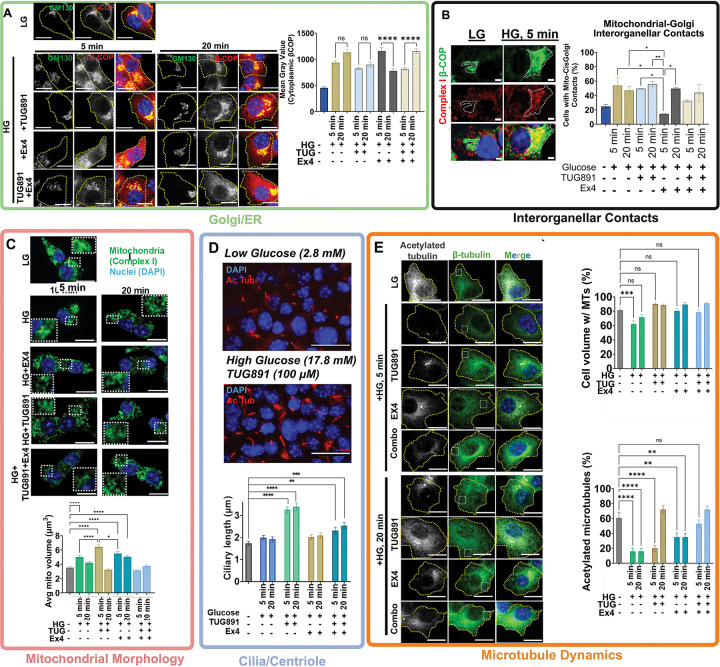
Differential GPCR and glucose stimulations lead to differential effects on cellular compartments based on live and fixed cell imaging. Changes in key cellular compartments with different stimulation conditions were assessed via IF and imaged via confocal microscopy at 60x magnification. Samples were collected at 5min and 20min after stimulation. The following compartments were assessed, with representative images and image quantification shown for each: (**A**) Golgi and vesicular traffic machinery morphology and spreading with dotted lines indicated cell borders (magenta: β-COP; cyan: GM130; blue: DAPI) (scale = 10μm), and quantification of mean gray value cytoplasmic β-COP was assessed for each condition (far right); (**B**) mitochondria-Golgi inter-organellar contacts, with dotted lines outlining the Golgi border to reflect whether mitochondrial are excluded (left, low glucose) or overlapping with Golgi (right, high glucose 5min) (blue: DAPI; green: β-COP; red: Complex-I)(scale = 10μm); quantification on left showing what percentage of cells in each condition show evidence of overlap with Golgi, with error bar= SEM(**C**) mitochondrial morphology, with blown up insets showing representative regions highlighting how tubular/fragmented the mitochondria are in that condition (green: Complex 1) (scale = 10μm), with average mitochondrial volume being assessed for each condition (below); (**D**) ciliary lengthening (red: acetylated tubulin; blue: DAPI) (scale = 20μm), with graph quantifying average length of cilia in each condition; (**E**) total microtubule density (green: β-tubulin) versus acetylated microtubules and yellow lines marking cell borders (white: acetylated tubulin; blue: DAPI) (scale = 10μm); differences in density were measured as % cell volume encompassed by total tubulin (upper right) versus acetylated tubulin (lower right); Experiments were performed in biological replicate, with ~100 cells per replicate except for mitochondrial volume which was >2000 mitochondria per condition and ~20 cells per condition for β-COP. Error bars= SEM. Statistical significance was assessed via One-Way ANOVA, with *P = 0.05; **P = 0.01; ****P = 0.0001. Plots were generated using GraphPad Prism software.

Several specific organelles exhibited dynamic yet stimulus-specific adaptation. For example, we assessed changes in dynamics in Golgi and membrane traffic machinery in different GSIS treatment conditions. All stimulation conditions led to increased cytoplasmic staining of β COP (a marker for COP I vesicles) and expansion away from the cis-Golgi, as marked by GM130. This is consistent with an increase in remodeling of the Golgi and mobilization of secretory machinery in response to increased metabolic demand upon glucose treatment. While TUG891 staining patterns were consistent with high glucose treatment alone, Ex4 stimulation led to an early increase in β COP cytoplasmic staining (Fig. 5A). Interestingly, we observed a similar trend in differential Ex4 membrane traffic dynamics in the context of mitochondrial-Golgi contacts, as Ex4 showed a marked delay in establishing these transient contacts (Fig. 5B). Taken together, these findings demonstrate that glucose and distinct GPCR signals recruit specific intracellular machinery and remodeling pathways to potentiate insulin secretion, underscoring the stimuli-specific compartmentalization of signaling in β cells.

We stained for mitochondria to assess changes in overall morphology, as these highly dynamic organelles frequently change in motility and morphology in response to nutrient status of the cell.^40,41^ We stained for Complex I, a key marker of the outer membrane of mitochondria which serves as a key regulator of the electron transport chain. We observed an increase in individual average mitochondrial volume at 5min (which we will term here as “elongation”) after stimulation, consistent with either an increase in mitochondrial fusion or a decrease in mitochondrial fission.^42^ The pattern of mitochondrial dynamic fission/fusion states changes, though, leading to decreased mitochondrial volume back to baseline by 20min either due to increased fission or decreased fusion. Notably, while Ex4 led to a prolonged “elongated” state compared to other stimuli, Combo treatment showed less increase in average individual mitochondrial volume compared to other conditions (Fig. 5C). We do note that there appear to be selective phosphorylations of mitochondrial GPCRs compared to high glucose that may contribute to ligand-specific changes in mitochondrial functionality/morphology. For example, we observe that the putative PKA substrate PFKL[S775] (ATP-dependent 6-phosphofructokinase) has selectively increased phosphorylation compared to high glucose or TUG891 treatment. This phosphorylation site is known to turn on the kinase catalytic activity which is required for committing to the first step of glycolysis,^43^ posing interesting questions as to potential differential effects on metabolic function. Furthermore, we see increased phosphorylation of PKA substrate Trafficking kinesin-binding protein TRAK1[S716] (a critical regulator of mitochondrial motility) upon GPCR stimulation, consistent with our data showing altered mitochondrial dynamics upon receptor stimulation.

Strikingly, HG+TUG891 combination stimulation triggered rapid and pronounced ciliary elongation within 5 minutes, which persisted for over 20min (Fig. 5A), whereas no evident changes were observed in glucose- or Ex4-treated groups. Cells treated with the Combo condition also exhibited a significant reduction in ciliary lengthening compared to HG+TUG891, suggesting competition or negative cross-regulation of GLP1-R on FFAR4 signaling. These findings align with our phosphoproteomic data indicating enrichment of FFAR4-regulated ciliary phosphoproteins, including the sodium-bicarbonate co-transporter SLC4A7[S960]^39^ (Fig. 2E).

We noted a distinct pattern in microtubule remodeling and PTMs during GSIS. The abundance of total assembled tubulin (assessed by IF of β tubulin) only showed modest fluctuation, with high glucose leading to the greatest degree of microtubule disassembly, especially along the cell periphery (Fig. 5E). This is consistent with previous reports suggesting that peripheral microtubule disassembly upon high glucose treatment is important for insulin secretion.^44^ We considered the hypothesis that microtubule disassembly might be linked to microtubule stabilization by acetylation, well-known to modify highly stable microtubules in primary cilia.^45–47^

Staining for acetylated tubulin revealed striking differences between the various GPCR ligands over time. HG or Ex4 stimulation led to persistent microtubule deacetylation at 5- and 20-minutes post-treatment, with HG having the strongest overall reduction in acetylated microtubules. However, in the case of Ex4, the preserved acetylated microtubule pool is strongly localized to the cis-Golgi. This is consistent with previous works demonstrating that the Golgi microtubule organizing center (MTOC) serves as the primary nucleation site for β cells, compared to undifferentiated, cycling cells where the centrosome is the primary MTOC.^44,48,49^ While TUG891 stimulation condition also led to a strong decrease in acetylated microtubules at 5min, we observed a rapid recovery of acetylated microtubules at 20min. Interestingly, the combination treatment leads to reduced microtubule deacetylation at 5min compared to the other treatments and a recovery of acetylated microtubule network by 20min post-stimulation. We hypothesized that microtubule acetylation control could be a direct consequence of altered phosphorylations of key regulatory machinery.

### Phosphorylation of ATAT1 and HDAC6 orchestrates microtubule remodeling

**Figure 6:**
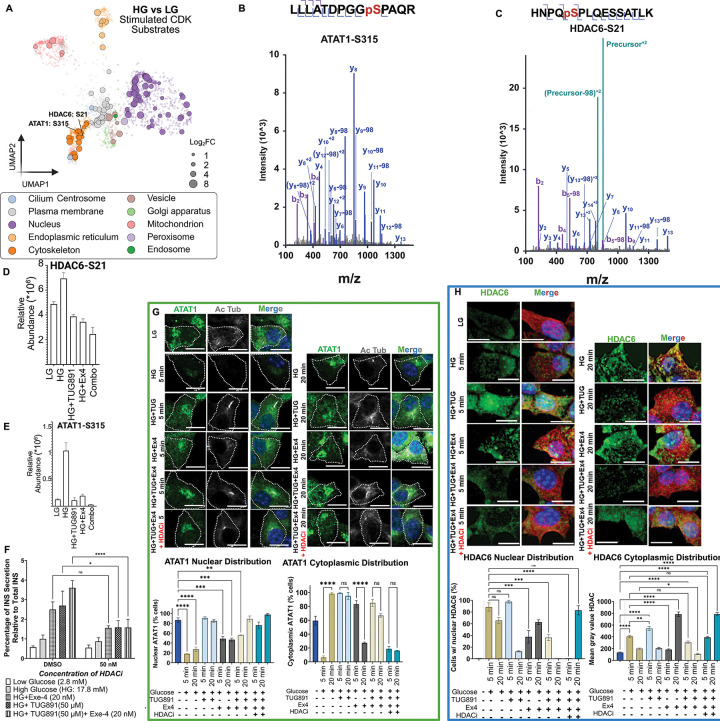
Differential HDAC6 and ATAT1 phosphorylation serve a pivotal role in orchestrating context-specific microtubule stability and dynamics during GSIS. (**A**) UMAP comparing high glucose to low glucose phosphorylations in MIN6 cells revealed key phosphorylations of known acetylation/deacetylation pathway regulators: HDAC6[S21] and ATAT1[S315] (both of which phosphorylations are conserved in humans). (**B-C**) Representative spectra of HDAC6[S21] (**B**) and ATAT1[S315] (**C**) phosphorylations as derived from Skyline software. To the top of each graph is the amino acid sequence for the peptide of interest, with the phosphorylated site indicated in red font with a “p” in front. (**D-E**) Plots showing the relative abundance (×10^6) of each phosphorylation in the following different GSIS treatment conditions in MIN6 cells, 15 minutes after stimulation: LG, HG, HG+TUG891, HG+Exendin4, and HG+TUG891+Exendin4 (Combo). Error bars= SD, N=3 (**F**) Plot measuring the effects of HDAC6 inhibitor treatment (5nM Ricolinostat (ACY-1215)) on percentage insulin secretion as measured by luciferase assay in MIN6 cells. Cells were grown in low glucose and preincubated either with DMSO or HDAC6i. Samples were treated with one of the following conditions for 30 minutes before harvesting supernatant (without washout of DMSO or HDAC6i): LG, HG, HG+TUG891, HG+Exendin4, or HG+TUG891+Exendin4 (Combo). Error bars=SD, N=3. (**G**) Analysis of HDAC6 localization via IF of MIN6 cells at different time points after stimulation of GSIS conditions. Above are shown confocal z-stack images (60x magnification) were collected, with green= HDAC6, red= β-tubulin, blue= DAPI. Scale = 10μm. Below are plots showing quantification of HDAC6 localization at nucleus (left) versus cytoplasm (right) under different treatment conditions. Error bars= SEM. Statistical significance was assessed via One-Way ANOVA, with *P = 0.05; **P = 0.01; ***P=0.001; ****P = 0.0001. Plots were generated using GraphPad Prism software. (**H**) Analysis of ATAT1 localization via IF of MIN6 cells at different time points after stimulation of GSIS conditions. Above are shown confocal z-stack images (60x magnification) were collected, with green= ATAT1, white= acetylated tubulin, blue= DAPI. Scale= 10μm. Below are plots showing quantification of ATAT1 localization at nucleus (left) versus cytoplasm (right) under different treatment conditions. Error bars= SEM. Statistical significance was assessed via One-Way ANOVA, with *P = 0.05; **P = 0.01; ***P=0.001; ****P = 0.0001. Plots were generated using GraphPad Prism software. (**I**) Imaging of MIN6 cells treated with different GSIS stimulating conditions reveals context-dependent differences in frequency of mitochondria-cis Golgi inter-organellar contacts. Cells were stained with the following markers: Complex I to mark mitochondria (red), -COP to mark (green), and DAPI to mark nuclei (blue). Z-stacks were collected via confocal microscopy at 60x magnification. Scale bar= 10μm. Shown here are representative images of low glucose versus high glucose (5min) treatments. (**J**) Plot showing quantitation of percentage of cells with mitochondria-cis Golgi contacts under different GSIS stimulation conditions, at 5 versus 20min timepoints post-stimulation. Error bars= SEM. Statistical significance was assessed via One-Way ANOVA, with *P = 0.05; **P = 0.01; ***P=0.001; ****P = 0.0001. Plots were generated using GraphPad Prism software.

To link phosphorylation dynamics with organelle remodeling during insulin secretion, we focused on phosphoproteins with well-established regulatory sites, notably ATAT1 and HDAC6. These enzymes regulate microtubule stability by controlling the reversible acetylation of α-tubulin.^50^ Phosphorylation of ATAT1[S315] promotes α-tubulin acetylation on Lys-40 and microtubule stabilization^51,52^ whereas phosphorylation of HDAC6[S21] enhances its deacetylase activity, driving microtubule deacetylation and depolymerization^53^. Our phosphoproteomics analysis identified ATAT1[S315] and HDAC6[S21] as prominent, glucose-induced cytoskeletal phosphosites (Fig. 6A–C), with markedly higher levels under glucose compared to other conditions (Fig. 6D–E). Functionally, acute treatment with the selective, FDA-approved HDAC6 inhibitor Ricolinostat at IC significantly blunted insulin secretion specifically in GPCR agonist regulatory conditions compared to glucose treatment alone (Fig. 6F; Fig.S3), highlighting a critical role for ATAT1/HDAC6 dual regulation in β cell secretory responses.

Immunofluorescence further revealed distinct subcellular dynamics. ATAT1 was broadly distributed throughout MIN6 β cells, with strong colocalization with the cis-Golgi apparatus (as marked by GM130) (a novel localization, as ATAT1 has only ever been seen to localize to Golgi in sperm^54^) (Fig. 6G, Fig. S4). Glucose stimulation triggered rapid nuclear export of ATAT1 within 5 minutes, persisting through 20 minutes (Fig. 6G, bottom panel, Fig.S5). GPCR agonists produced variable effects, with Ex4 causing modest nuclear export, while TUG891 or the combination elicited little change (Fig. 6G, bottom panel, Fig. S5). These findings are consistent with reports linking [S315] phosphorylation to nuclear exit, as previously described.^51^ Interestingly, receptor treatment led to differential ATAT1 distribution outside of the nucleus, with ATAT1 having increased Golgi localization and only transient cytoplasmic localization upon Ex4 treatment, and ATAT1 having increased and enduring cytoplasmic distribution upon TUG891 treatment.

In contrast, HDAC6 displayed dynamic changes in both abundance and localization. Baseline levels were low, but all stimulation conditions robustly upregulated cytoplasmic HDAC6, independent of inhibitor treatment (Fig. 6H; Fig.S6). HDAC6 localized to both nuclei and cytoplasm, where it appeared in puncta adjacent to β-tubulin. Under stimulatory conditions (*e.g*., HG 20min, TUG 5min, Ex4 20min, Combo 5min), HDAC6 showed striking colocalization with β-tubulin, coinciding with reduced acetylated microtubules (Fig. 6H; Fig.S6).

Collectively, these results demonstrate that glucose and GPCR agonists engage ATAT1 and HDAC6 through opposing phosphorylation events to orchestrate β cell microtubule remodeling, thereby linking cytoskeletal dynamics to insulin secretion.

### Shared and divergent phospho-signaling programs in human and mouse β cells

**Figure 7:**
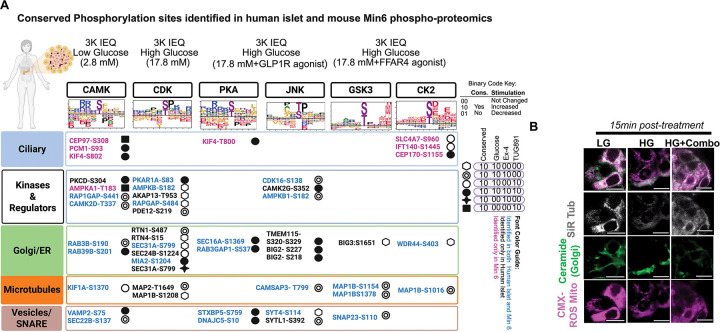
Human cadaveric islets and mouse MIN6 cells show commonalities in changes in cellular function and phosphoproteomic signatures upon different GSIS treatment conditions. (**A**) Table showing representative phosphorylations detected from human pancreatic islet (3,000 IEQ per condition). Font color indicates which GSIS phosphorylations are shared between human cadaveric islets and mouse MIN6 stable cell lines (pink= MIN6 only, black= human islet only, blue= shared). Phosphorylations are sorted by row based on known cellular compartment and sorted by column based on predicted regulatory kinase (along with the kinase motif analysis below). Dots next to the phosphorylation reference the binary code plot on the right, which highlights the differential abundance depending on experimental conditions. (**B**) Representative live cell imaging panels of human cadaveric islets showing changes in ciliary length (left) and microtubule dynamics (right) upon different stimulation conditions. Images were collected via confocal timelapse imaging at 40x magnification, collecting z-stacks every ~5min using far red SiR-Tubulin live cell imaging dye. Scale bar= 10μm.

To understand the degree to which the GSIS phosphoproteome that we identified in mouse β cells is conserved in human tissue, we have mapped glucose and GPCR induced phosphorylation in cadaveric human islets from healthy patients. The phosphoproteome of human islets was profiled 15min after stimulation under four conditions, (1) LG, (2) HG, (3) HG+Ex4, and (4) HG+TUG891 (after confirming islet health and reproducibility of our findings in MIN6 using perifusion assay for insulin secretion (Fig.S7)). We detected more than 5,000 phosphorylation events on 1,800 proteins (threshold, FDR<0.01). The somewhat smaller number of phosphorylations is due to the smaller sample size due limitations in available patient islet samples. Compared to LG baseline, we observed increased phosphorylation levels in HG, HG+Ex4, and HG+TUG891 (Supplementary Table 1,2, Fig. 7A), including kinases, calcium regulatory factors, Golgi/ER proteins, MT-associated proteins, and secretory vesicle factors/SNAREs. Remarkably, >80% of phosphorylation sites induced by glucose or GPCR agonist were conserved in the phosphoproteome from MIN6 cells; examples of key conserved phosphorylation sites are shown in Fig. 7A. In some cases, like DNAJC5[S10] and STXBP5[759], phosphorylation levels increased in HG and were further increased by the addition of GPCR agonists (Supplementary Table 1,2). In other cases, we observed agonist-specific phosphorylation. For example, levels of microtubule MAP2[T1649] phosphorylation were induced by HG and in HG+Ex4 but not HG+TUG891. Conversely, elevation of several phosphopeptides were detected in human islets but not MIN6 cells (Fig. 7A, Supplementary Table 1,2). This could reflect species specificity, differing β cell responses in whole islets versus cultured β cells but could equally as liked reflect contamination with phosphoproteins from other islet cells (*e.g*., α cells), or simply technical dropouts for less abundant proteins. Collectively, these phosphoproteomics studies in human islets identified multiple conserved glucose- and GPCR-dependent responses in cells.

To determine whether phosphoproteomic changes were mirrored by subcellular organelle remodeling, we assessed microtubules, Golgi, and mitochondria in human islet cells via live cell imaging. Consistent with the conservation in phosphoproteomics, human islets displayed reduction in microtubule density following glucose stimulation (Fig. 7B, Fig.S8). In addition, we observed the same trends in mitochondrial fragmentation in the first 5min followed by lengthening ~20min, with combined GPCR stimulation enhancing the speed/degree of mitochondrial elongation (Fig. 7B, Fig.S8). These findings underscore conserved molecular and organellar responses across mouse and human β cells to glucose and GPCR stimulation.

## DISCUSSION:

Here, we deployed STAMP, a newly-developed workflow integrating phosphoproteomics, microscopy, and functional assays, to investigate molecular mechanism by which glucose and GPCR signals regulate insulin secretion in β cells. We revealed that thousands of phosphorylation events occur within minutes of stimulation, but the patterns differ depending on whether cells respond to glucose or to GLP1-R and FFAR4 agonists, or in combination of all three stimuli. Glucose primarily drives phosphorylation changes in cilia, mitochondria, microtubules, and the Golgi, while GPCR agonists reshaped signaling in overlapping but also unique compartments, producing both synergistic and attenuated effects when combined. Targeted analysis of canonical pathways revealed stimulus-specific mechanisms: GLP1-R uniquely activated the ERK cascade, while PKA signaling specificity was refined by phosphorylation of the PKA-AKAP-PDE complexes at distinct subcellular sites. Significantly, a selective kinase-inhibitor screen identified over 17 specific kinases important for induced phosphorylations and linked these phosphorylation programs to functional insulin release, confirming PKA, CAMKII, and other kinases as critical nodes. Live and fixed cell imaging further demonstrated that phosphorylation dynamics translated into a wide variety of β-cell architecture remodeling, including TUG891-induced ciliary elongation, glucose-dependent microtubule disassembly, robust stimulus-dependent Golgi remodeling, dynamic mitochondrial fusion-and-fission, and previously unrecognized dynamics in Golgi-mitochondrial contacts. Candidate-based mechanistic analysis of ATAT1 and HDAC6 further illustrated how an opposing phospho-regulated enzymes directly control microtubule acetylation and in turn insulin secretion. Finally, conserved phosphorylation and organelle responses in human islets support the physiological relevance of these findings. Together, our work provides an unprecedently comprehensive and compartment-resolved view of how metabolic and GPCR cues orchestrate an intricate balance of β cells signaling network to fine-tune insulin secretion.

Our findings highlight that insulin secretion is governed by a dynamic architecture of compartmentalized and stimulus-specific signaling programs that sometimes intersect, rather than by a few parallel canonical pathways. Our integrated STAMP analyses provided mechanistic insights into how such complexity is orchestrated through substrate specificity of kinase recognition motifs, spatial compartmentalization by scaffold proteins, engagement of distinct subcellular organelles, temporal signaling dynamics, as well as regulatory feedback. Glucose stimulation alone triggered broad phosphorylation changes across multiple organelles, particularly in the cytoskeleton, cilia, mitochondria, and Golgi, underscoring its central role as a metabolic driver of β cell response. In contrast, GPCR agonists (GLPA1R and FFAR4) elicited partially overlapping yet distinct phosphorylation signatures, engaging signaling modules not activated by glucose. For example, GLP1-R selectively activated the ERK cascade, while FFAR4 uniquely modulated mitochondrial AKAP/PDE complexes and promoted rapid ciliary elongation. These differences suggest that while glucose establishes the basal framework for insulin secretion, GPCR pathways superimpose modulatory layers that fine-tune both the spatial and temporal aspects of signaling. Importantly, the attenuation of hundreds of phosphorylation events in the combined agonist condition may point to regulatory crosstalk and feedback that may prevent excessive or maladaptive signaling in the β cell (either because of a delay in phospho-signaling or suppressive phosphatase activity to delay the feedback signal). Such a feedback framework is evident in other aspects of biology, such as GABAergic suppression to increase neural excitability or hyperphosphorylation of Rb to drive cell cycle progression.^55–58^ Note, though, that this model would require further testing with higher temporal resolution to effectively assess whether the suppressed phosphorylation at the 20min time point is truly indicative of altered feedback loop timing upon co-stimulation compared to individual stimulations alone. It would also be interesting to note whether this putative feedback loop is specific for FFAR4-GLP1-R mediated intercommunication, or if it applies to other receptor pairs. Collectively, these discoveries provide context for understanding how β cells achieve both robustness and flexibility in insulin secretion. Moreover, this compartmentalized, stimulus-specific architecture may also help explain why different pharmacological agents targeting GPCRs yield additive or synergistic benefits in enhancing β cell function, offering new opportunities for therapeutic strategies targeting precise modulation of β cell insulin secretion.

This study highlights the utility of STAMP as a strategy for deconvolving transient molecular signals and changes in cellular function that drive pancreatic β cell insulin secretion. By combining the power of homogeneous cell populations responding to stimuli with time-resolved imaging and phosphoproteomics, we establish a system where one can link molecular changes to modifications in the cellular landscape. This can aid in identifying underlying signals that orchestrate the cellular events driving healthy β-cell GSIS. Though previous studies have explored the dynamics of context-specific fluctuations in phosphorylations during GSIS,^1,2^ there is still much to be studied to bridge the gap between spatial context and the mechanisms driving it. We add yet another layer of complexity to studying the GSIS response through A) stimulating enhanced insulin secretion by activating GPCRs FFAR4 and GLP1-R alone or in combination to track changes in organelle dynamics, B) assessing the context specificity of kinase signaling for high glucose versus receptor treatment during GSIS, and C) deciphering how different stimuli can operate through much of the same types of machinery but in a compartment-specific manner (*e.g.*, TUG891 specifically upregulating phosphorylation of mitochondrial AKAP1[S103] while Exendin-4 leads to upregulated ERK1[Y204] phosphorylation, the classic “ON” signal).^59^ By studying both time-resolved spatial and molecular context, we can start the much-needed work of identifying how exactly different receptor stimulations and particular branches of signaling pathways differentially modulate GSIS. This is especially critical, as our data show that combined stimulation of FFAR4 and GLP1-R during GSIS leads to enhanced insulin secretion after 30min compared to any individual treatment alone (Fig. 2A). We observed key receptor-specific phosphorylations that are suggestive of different compartmentalization of signaling, and we observed correlational changes in these compartments based on IF. For example, it is interesting to note that there are number of phosphorylations of known ciliary machinery that are suppressed specifically upon FFAR4 stimulation, including KIF3A[S689], Cep170[S872], Cep97[S469]. On the other hand, several vesicular traffic pathway proteins (*e.g.*, Sec22B[S137], MIA2[S1131], SNAP23[S110], STXBP5[S759]) are specifically upregulated upon GLP1-R stimulation. Based on general IF of cellular compartments, many of the strongest changes in cilia occurred upon FFAR4 stimulation and the strongest changes in vesicular traffic machinery occurred with GLP1-R stimulation. One case study that we used to see whether the fluctuations in key proteins can be linked directly to cellular phenotypes was in the context of the ATAT1 and HDAC6 node, critical regulators of microtubule acetylation. Previous studies have noted the pivotal role that microtubules play during GSIS, with their dynamic rearrangement facilitating insulin granule release.^44,48^ Other works have shown that these microtubules are naturally very short, which may contribute to their ability to dynamically rearrange and uncage microtubules based on signaling context. Yet, the mechanisms that regulate microtubule dynamics in this context have not been fully elucidated. Our observation via IF that receptor stimulation suppresses microtubule de-acetylation and de-stabilization (especially in the case of FFAR4), coupled with phosphoproteomics demonstrating decreased phosphorylation upon receptor stimulation compared to high glucose treatment alone, supported a model in which microtubule acetylation plays a key role in mediating GSIS (Fig. 6). Based on IF and inhibitory treatment studies, we confirmed differential localization patterns of each of these factors based on receptor stimulation. Together, one can propose a hypothesis in which HDAC6[S21] (either directly or indirectly) promotes its deacetylation function while ATAT1[S315] phosphorylation suppresses its pro-acetylation function. Note that while the HDAC6[S21] phosphorylation has no known function, ATAT1[S315] has previously been reported to induce nuclear exit, consistent with our data. Further studies will need to be done to identify the exact function of these phosphorylations, as well as the role of regulated microtubule acetylation in modulating GSIS. One possibility is that the acetylation dynamics contribute to differential microtubule stability that could thus support increased vesicular traffic during the second wave because of the pre-existing cytoskeletal networks for traffic. Another possibility could be similar to the context of neurons, in which acetylation increases the processivity of motor proteins enabling enhanced kinesin and dynein traffic.^60^ Perhaps contextual increased vesicular processivity enables enhanced secretion of insulin granules. Further testing will be required to distinguish between these two models.

Our work also reveals new insight into the fundamental cell biology underlying β cell GSIS and opens exciting new directions to explore. Though there are many unanswered questions that have yet to be pursued, one interesting angle to study is the concept of the Golgi as a critical nexus for β cell function-namely because of its inter-organellar contacts with the microtubule cytoskeleton. Previous studies have established that the Golgi apparatus is the central MTOC for pancreatic β cells rather than centrosomes.^61^ This is consistent with the literature ascribing that the site for microtubule nucleation shifts as cells exit the cell cycle and assume cell fate, shifting away from centrosomal to Golgi-associated nucleation. It is interesting to note, though, that while both FFAR4 and GLP1-R lead to faster recovery of microtubule acetylation compared to high glucose stimulation alone, FFAR4 stimulation appears to favor acetylation of centrosomal microtubules while GLP1-R stimulation favors Golgi-associated microtubules. This draws to question what is the mechanism by which FFAR4 and GLP1-R differentially regulate the HDAC6/ATAT1 signaling node. Neither FFAR4 or GLP-1R stimulation induce enhanced HDAC6 or ATAT1 phosphorylation, so there is likely some other mechanism at play here that provides compartmentalization of microtubule regulation. It would be interesting in the future to assess whether other ciliary receptors also specifically regulate centrosomal microtubule dynamics. Our data also show, for the first time to our knowledge, that ATAT1 strongly localizes to the Golgi in all β-cell conditions. Previous studies indicate that ATAT1 normally localizes to either the nucleus or the cytoplasmic space in most other cell types studied (including in vesicles in axons)^60^ except for in the case of sperm where it also showed Golgi localization.^54^ Future studies should be devoted to understanding the differential, compartment-specific functions of ATAT1 in diverse cell types. We do note, though, an interesting change in the dynamic localization pattern of ATAT1 in cytoplasmic/vesicular pools versus cis-Golgi pools. Future work will be devoted to mapping these distributions over time to see how these trends correlate with the two waves of insulin secretion and how GPCR stimulation modulates them. We also observed highly transient, context-specific inter-organellar contacts between mitochondria, cis-Golgi, and vesicular traffic machinery (Fig. 5E). Relatively little is known about the function of mitochondrial-Golgi and mitochondria-Golgi derived vesicle contacts compared to more commonly studied mitochondrial-ER contacts, yet it is believed that these highly transient sites are important for modulating membrane composition (more specifically of PI4P phospholipids, important for these inter-membrane compartment contacts^62^), calcium signaling, mitochondrial fission/fusion dynamics,^63^ and efficient energetics.^64,65^ Beyond our live and fixed cell imaging showing GPCR-modulated mitochondrial-Golgi contacts, we also observed key phosphorylations of mitochondria-Golgi contact regulatory proteins including oxysterol-binding protein (OSBP).^66^ Notably, these contact sites seem to be prolonged upon inhibition of HDAC6. Future work to decipher the function and dynamics of these contacts in the context of GSIS, as well as the role of microtubule regulation with relation to these sites.

Overall, this study advances our understanding of β cell biology by providing context-specific regulation of GSIS. Findings reported here have generated a significant resource to formulate hypotheses and inform future studies of mechanisms underlying cellular phenotypes during GSIS. Based on these results, we see how differential GPCR stimulation leads to context-specific changes in temporal-spatial regulation of post-translational modifications in regulating GSIS function. We extended the work further by repeating these studies in human pancreatic islets, identifying a high degree of overlap between the two datasets. These works highlight the value of in vitro and ex vivo model systems to probe fundamental mechanisms driving islet biology, with the goal of eventually applying these findings to finding therapeutic targets that address patient-specific disease lesions. Much future work will be needed, but these studies pave the way for detailed temporal-spatial mapping of the two waves GSIS and how GPCR modulation helps to calibrate these cellular responses.

### Lead contact

Further information and requests for resources and reagents should be directed to and will be fulfilled by the Lead Contact, Peter Jackson (pjackson@stanford.edu).

### Materials availability

All unique/stable reagents generated in this study are available from the Lead Contact with a completed Materials Transfer Agreement.

### Data and code availability

The published article includes all datasets generated during this study.

## METHODS:

### Reagents, antibodies, and plasmids

The following primary antibodies were used in this study. **For IF**: acetylated tubulin (mouse IgG2B; Santa Cruz; Cat #: SC-23950; 1:2000 of 200 μg/ml stock), -tubulin (mouse IgG1; Sigma-Aldrich; Cat #: T5201), insulin (guinea pig; discontinued antibody gifted from Seung Kim Lab;^68,69^ 1:500 of stock (mouse IgG1; Sigma-Aldrich; Cat #: T5201; 1:2000 of 2mg/mL stock), GM130 (mouse IgG1; BD Biosciences; Cat #: 610823; 1:500 of 250μg/mL), -COP (rabbit; gifted from Richard Kahn lab;^70^ 1:1000 of stock), HDAC6 (Rabbit; US Biologicals; Cat #: H1827-66; 1:250 of 0.1mg/mL stock), ATAT1 (Rabbit; Millipore-Sigma; Cat #: HPA046816; 1:250 dilution of 0.1mg/mL stock), Complex I (mouse Ig2B; Thermofisher; Cat #:43-8800; 1:1000 of 1mg/mL stock). **For Western blot**: pERK 1/2 (mouse IgG1; Cell Signaling Technologies; Cat #: 9107S; 1:2000 dilution), Lamin (rabbit IgG1; Cell Signaling; Cat #:2032S; 1:1000 dilution of 0.5mg/mL stock), and -actin rabbit IgG; Cell Signaling; Cat #: 8457S; 1:2000 of 1mg/mL stock).

The following Alexa Fluor secondary antibodies (Invitrogen) were used in this study: (i) Donkey-anti-Rabbit 488 (Cat #: A-32790), (ii) Donkey-anti-Rabbit 568 (Cat #: A-10042), (iii) Donkey-anti-Rabbit 647 (Cat #: A-21447), (iv) Goat-anti-mouse IgG1 488 (Cat #: A-21121), (v) Goat-anti-mouse IgG1 568 (catalog no. A-21124), (vi) Goat-anti-mouse IgG1 647 (Cat #: A-21240), (vii) Goat-anti-mouse IgG2a 488 (Cat #: A-21131), (viii) Goat-anti-mouse IgG2a 568 (Cat #: A-21134), (ix) Goat-anti-mouse IgG2a 647 (Cat #: A-21241), (x) Goat-anti-mouse IgG2b 488 (Cat #: A-21141), (xi) Goat-anti-mouse IgG2b 568 (Cat #: A-21144), (xii) Goat-anti-mouse IgG2b 647 (Cat #: A-21242)

The following drugs were used in this study: Exendin-4 (Cayman; Cat #: 11096), TUG891 (Tocris Bioscience; Cat #: 4601), Ricolinostat (HDAC6 inhibitor) (MedChem Express; Cat #: HY-16026), H-89 (PKA inhibitor) (Sigma-Aldrich; Cat #: B1427). Sterile DI water or DMSO (Sigma-Aldrich; Cat #: 276855) was used for diluting drugs. The kinase inhibitor screen (240 inhibitors tested) was shared with us by the Nathanael Grey lab^32–34^ in 96-well plate format (https://graylab.stanford.edu/probe-resources/) with the Stanford High-Throughput Screening Core (Stanford HTS).

The following reagents are used for phosphoproteomics: Halt^™^ Protease Inhibitor 100x (Thermofisher Scientific; Cat #: 78429), Halt^™^ Phosphatase Inhibitor 100x (Thermofisher Scientific; Cat #: 78420), 2-Chloroacetamide (CAA) (Sigma-Aldrich; Cat #: C0267-100G), HEPES, >99.5% Titration (Sigma-Aldrich; Cat #: H3375-5KG), Blunt-End Needles, 16 Gauge (STEMCELL; Cat #: 28110), Acetonitrile (ACN) (Honeywell; Cat #: 14261-1L), Sodium Chloride (NaCl) (Cat #: S3014-5KG, Sigma-Aldrich), Methanol (Millipore Sigma; Catalog #: 900688-1L), Water, Optima^™^ LC/MS Grade (Fisher Chemical; Cat#: W6-4), Trypsin/Lys-C Mix, Mass Spec Grade (Cat #: V5073, Promega), ProteaseMAX (Promega; Cat #: V2072), Ammonium Bicarbonate (ABC) (Honeywell; Cat #: 40876-50G), Pierce^™^ Premium Grade TCEP-HCl (Thermofisher Scientific; Cat #: PG82080), Pierce^™^ BCA Protein Assay Kit (Thermofisher Scientific; Cat #: 23225), Pierce^™^ Bovine Serum Albumin Standard Pre-Diluted Set (Thermofisher Scientific; Cat #: 23208), Pierce^™^ Quantitative Colorimetric Peptide Assay (Thermofisher Scientific; Cat #: 23275), High Select Fe-NTA Phosphopeptide Enrichment Kit (Thermofisher Scientific; Cat #: A32992), Empore C18 47mm Extraction Disk, Model 2215 (Empore; Cat #: 320907D), Formic acid, 99.5%, Optima^™^ LC/MS Grade (Thermofisher Scientific; Cat #: A117-50), Trifluoroacetic acid (TFA) (Fisher Scientific; Cat #: AAL06374AC)

### Model system: Cell Culture and Human Cadaveric Islets:

Mouse pancreatic MIN6 β cells were gifted from Seung Kim’s lab at Stanford University. Pancreatic MIN6-6 cells were gifted from Wenbiao Chen’s lab at Vanderbilt University. Stable cell lines were cultured in DMEM medium (buffered with sodium bicarbonate (Gibco; 25080-094) containing 10% heat-inactivated fetal bovine serum (Gemini; Cat #: 100-106), 10mM HEPES (Gibco; Cat #: 15630080) 0.1% v/v β-mercaptoethanol (Invitrogen; Cat #: 21985023), and 1% pen/strep (Gibco; Cat #: 10378016). Cells were maintained at low passage (below passage 30) to avoid genetic drift and checked at least monthly for mycoplasma using Hoechst staining and immunofluorescence. For all imaging and mass spectrometry experiments, 5×10^6 cells were plated into 10cm dishes. For all insulin secretion assays, 1×10^5 cells were plated per well of a 96 well dish.

Human cadaveric islets were obtained through Stanford Diabetes Research Center’s Islet Research Core. These samples were harvested from de-identified human donors. These samples were harvested less than 15hr of cold ischemia time. Islets were spun down at 200 g (2–4 Minutes) to remove the culture medium, washed with ice cold 1x PBS, and spun down again at 200rcf. Samples were then incubated with 190μL 2.8mM Low Glucose for two hours at 37°C before use for future assays (described below). For mass spectrometry experiments, 3000 IEQ were plated for each condition. To induce stimulated GSIS in isolated human pancreatic islets, cells were treated with 310μL of the following conditions: 2.8 mM glucose (LG), 25mM glucose alone (HG), 25 mM glucose with the FFAR4 agonist TUG891 (161μM), or 25 mM glucose with Ex4 (32.2nM). Following drug addition, cells were incubated at 37 °C for 15min. After incubation, the culture medium was collected for subsequent ELISA. Cells were then lysed by addition of 300μL lysis buffer and immediately boiled for 5 minutes at 95 °C. Lysates were stored at −80 °C until further preparation for phosphoproteomic analysis.

### Experimental conditions for Min 6 experiments:

MIN6 cells were cultured and subjected to serum starvation in 5 mM glucose for 24 hours prior to stimulation. After preincubation with low glucose (2.7 mM) for one hour, cells were stimulated under the following conditions (each compared with low glucose control): High Glucose (HG: 17.8mM), HG + 20nM Ex4, HG + 100μM TUG891, HG + 20nM Exendin-4 + 100μM TUG891 (Combo), Combo + 10μM NKY-80 (ADCY inhibitor), Combo + 1μM H-89 (PKA inhibitor), Combo + 1μM KN-93 (CAMKII inhibitor), Combo + 1μM Silmitasertib (CK2 inhibitor). For the phosphoproteomics cells are lysed at 10 minutes. For the imaging cells stimulated for durations of 5 and 20 minutes as indicated, followed by sample harvest for downstream analysis.

### Kinase inhibitor screen on glucose-stimulated insulin secretion (GSIS) in MIN6 cells (Supplemental Table 3, Fig. S1)

MIN6–6 cells were first cultured under standard conditions and then seeded into 96 multiwell plates at 80% confluency for 24 hours to allow attachment and growth. After initial culture, cells were serum-starved for 24 hours at 5 mM Glucose to synchronize metabolic activity. To initiate the kinase inhibitor screen, cells were pre-incubated for 1hr in low glucose (2.8mM) medium with 5 μM of each compound from the Gray Lab kinase inhibitor library (240 drugs). Following this pre-treatment, cells were stimulated for 30min at high glucose (17.8 mM) in the continued presence of the kinase inhibitors, and 100μM TUG891 and 20nM Ex4, including low glucose and high glucose controls. After stimulation, glucose-stimulated insulin secretion was quantified using the Nano-Glo^®^ Luciferase Assay according to manufacturer instructions, with luminescence measured to assess GSIS across conditions.

### Phosphoproteomics of MIN6 and human cadaveric islets

Stock solutions were prepared, including 1 M tris-HCl (pH 8.5) and 5 M potassium hydroxide (KOH). Plates (10cm) containing 8 × 10^6 MIN6 cells were harvested for each replicate and time point for phosphoproteomic analysis. SDC lysis buffer was prepared fresh using 4% (w/v) SDC, 100mM tris-HCl (pH 8.5), and 1× Halt protease and phosphatase inhibitor cocktail. The buffer was made fresh. A 1ml volume of lysis buffer was added to each plate. Lysates were immediately heat-treated for 5min at 95°C to facilitate lysis and inactivate endogenous proteases and phosphatases. Then, lysates were homogenized by sonication at 4°C.

Reduction/alkylation buffer included 100mM TCEP and 400mM CAM, pH 7–8 adjusted with KOH, prepared immediately before use to preserve CAM activity. Then, disulfide bonds and carbamidomethylated cysteine residues were reduced by adding a 1:10 volume of reduction/alkylation buffer to the samples. Samples were incubated for 30min at room temperature at 1000 rpm, in dark. After removing the samples from the shaker, Lys-C and trypsin enzymes were added at a 1/100 ratio of protein/enzyme ratios, and the samples were digested for 16hrs at 37°C with shaking at 1000 rpm.

Following overnight digestion, peptides were acidified with TFA, centrifuged, and cleaned up using Sep-Pak tC18 1 cc columns as previously described. The purified peptides were then quantified using the ThermoFisher Pierce^™^ Quantitative Peptide Assay to ensure accurate input amounts for downstream enrichment. Subsequently, phosphopeptides were enriched using the High Select Fe-NTA IMAC phosphopeptide enrichment kit (ThermoFisher) following the supplier’s instructions for binding, washing, and elution steps.

Homemade Stage-Tips were constructed using two C18 Empore disks, following established procedures. The fabricated Stage-Tips were washed two times with 100μL of methanol, one time with 100μL of 80% acetonitrile/0.1% acetic acid, and two times with 100μL of 1% acetic acid. Enriched phosphopeptides were loaded onto the Stage-Tips in 100μL of 1% acetic acid. Subsequently, the Stage-Tips were washed three times with 100μL of 1% acetic acid to remove salts. Last, the phosphopeptides were eluted from the Stage-Tips using two elution steps of 30μL each, with 80% acetonitrile/0.1% acetic acid as the elution buffer.

### Liquid chromatography setup

A nanoELute ultrahigh-pressure nanoflow chromatography system was used and directly coupled online with a hybrid trapped ion mobility spectrometry—quadrupole time-of-flight mass spectrometer (timsTOF Pro, Bruker) using a nanoelectrospray ion source (CaptiveSpray, Bruker Daltonics).

### Chromatographic conditions

The liquid chromatography was conducted at a constant temperature of 50°C, using a reversed-phase column (PepSep column, 10 cm by 150 μm ID, packed with 1.5μm C18-coated porous silica beads, Bruker) connected to the 10μm emitter (Bruker). The mobile phase consisted of two components: Mobile Phase A, comprising water with 0.1/2% formic acid/ACN (v/v), and Mobile Phase B, comprising ACN with 0.1% formic acid (v/v).

### Gradient elution

Peptide separation was achieved using a linear gradient from 2 to 33% Mobile Phase B within 60 min. This was followed by a washing step with 95% Mobile Phase B and subsequent re-equilibration. The chromatographic process maintained the flow rate at 400nL/min.

### MS acquisition

Samples were analyzed using the timsTOF HT mass spectrometer in DDA-PASEF mode. The TIMS elution voltage was calibrated linearly to obtain reduced ion mobility coefficients (1/K0) by using three selected ions from the Agilent ESI-L Tuning Mix [mass/charge ratio (m/z) 622, 922, and 1222]. The mass and ion mobility ranges were set from 100 to 1700 m/z and 0.7 to 1.3 1/K0, respectively. Both ramp and acquisition times were set at 100ms. Precursor ions suitable for PASEF-MS/MS were chosen from TIMS-MS survey scans using the PASEF scheduling algorithm. A polygon filter was applied to the m/z and ion mobility plane to prioritize features likely representing peptide precursors over singly charged background ions. The quadrupole isolation width was set to 2 Th for m/z < 700 and 3 Th for m/z > 700, with collision energy linearly increased from 20 to 60 eV as ion mobility ranged from 0.6 to 1.6 (1/K0).

### Mass Spectrometry Data Analysis

Raw data files were processed using MS Fragger software against the NCBI Homo sapiens RefSeq protein database. Search parameters included CID (collision-induced dissociation) fragmentation with a precursor error tolerance of 10 parts per million (ppm) and a fragment ion tolerance of 20 ppm. Searches included S/T/Y phosphorylation and up to three modifications per peptide besides standard modifications. Peptides were validated using Percolator and Protein Prophet at 1% FDR (false discovery rate). Protein quantification was performed using IonQuant, with normalization across runs and Match Between Runs settings accommodating retention time and ion mobility tolerances of 0.4 min and 0.05 (1/K0), respectively.^71^

Using the comprehensive kinase substrate specificity profiles established by Cantley’s lab (), we assigned the putative kinases for each phosphorylation site based on the tool available on PhosphoSite (Ref). The final kinase rankings were determined by calculating the percentile position of each kinase’s score within the distribution of scores generated from analyzing all available serine/threonine phosphorylation sites from PhosphoSite.^24^ The phosphorylation signaling maps are only plotted for the highly confident phosphorylations sites, manually inspected by Skyline. MS2 scans are extracted and confirmed for each phosphorylation site demarcated in the figures.

#### UMAP Analysis Based on localization with COMPARTMENT Database Annotation

All data processing and visualization were performed in Python (v3.11) using pandas, seaborn, and matplotlib, with an interactive interface built in Streamlit to facilitate dynamic data exploration and visualization.

Experimental phosphoproteomic data tables were analyzed with FragPipe-Analyst for the log_2_ fold change values and associated p-values. A background protein list containing all phosphoproteins served as the reference set for generating the UMAP embedding. This background enabled a standard coordinate space onto which experimental data were overlaid. Quantitative phosphoproteomics data were merged into a unified kinase-substrate-level table, consolidating all condition measurements per protein in a structured format. The COMPARTMENT database^72^ was employed to provide subcellular localization annotations for all proteins in the background protein list. This database integrates evidence from diverse sources, including experimental data, computational predictions, and literature text mining, to assign proteins to specific cellular compartments. These localization annotations enriched the biological interpretation of the UMAP embedding by linking proteomic changes to their respective subcellular contexts. Uniform Manifold Approximation and Projection (UMAP) was applied to the full background dataset to embed proteins in a two-dimensional space, preserving both local and global relationships among proteins. Condition-specific log_2_ fold change and p-value data were mapped onto the background UMAP embedding. Visualization outputs consisted of: Condition-specific protein data visualized on the UMAP coordinate space.

### Human Islet Secretion Assay and Hormone Assessment

The human islets were recovered for 2 hours in RPMI media (Thermo Fisher) supplemented 2.8 mM glucose (Sigma-Aldrich), 1mM Sodium Pyruvate (Thermo Fisher), 10mM HEPES (Thermo Fisher), and 0.1% (w/v) BSA (Sigma-Aldrich) prior to static insulin secretion analysis. Batches of 3000 IEQs were then treated with glucose or GPCR agonists as indicated for 15 min at 37 degrees. Supernatants were collected at the end of incubation and insulin levels were quantified by human insulin ELISA kits (Mercodia).

### Cryosectioning of human cadaveric pancreatic islets

After stimulation of different GSIS conditions, cells were fixed for 15 minutes with 4% PFA/PBS at 37°C. Samples were then spun down and washed 2x with PBS, spinning down and removing the supernatant from each wash. Using the Collagen Gel Cell Culturing Kit (FUJIFILM Wako; Cat #:638-00781), make a master mix of collagen components A and B (v:v = 8:1) enough for all tubes (usually 300μL per tube). Mix completely by vortexing. *It is recommended to use more AB mix than necessary at this point and remove the extra later.* Aliquot the AB mix to islet pellet and mix very well. Then, quick spin to collect islets at the bottom of the tube. Add component C (A:B:C = 8:1:1) to each tube, mix immediately by vortexing and then quick spin to sink islets to bottom. Collagen solidifies quickly at room temperature upon the addition of component C, so make sure to keep the samples on ice at all idle times. Incubate samples on ice for 10 minutes to allow the islets to immerse in the solution completely. Afterward, incubate the mix in a 37°C waterbath to allow the samples to solidify, ~30min. Use a pipet tip to poke the top of collagen and swirl gently to check whether the samples have solidified with the islets locked in place. Add 4% PFA/PBS to further fix for 30min-1hr at 4°C. Afterward, take off PFA to stop fixation and wash with cold PBS twice.

To prepare the samples for cryosectioning, pour the collagen chunk out (or pull out gently with tweezers) onto a petri dish. Cut off the extra collagen above the islet cluster. Then, put the small collagen piece back into the eppendorf tube and add in 30% sucrose. Incubate the samples overnight at 4°C or until the sample has sunk to the bottom of the tube.

After ensuring that the sample has sunk to the bottom of the tube, carefully take out the sample using tweezers onto a petri dish. Trim the collagen block using tweezers under a dissecting microscope to avoid losing sample. The purpose is to make the sample as small and flat as possible before embedding. Use a p10 pipette to remove residual liquid completely to avoid ice crystal formation during cryopreservation.

Use Sharpie to draw a line around the collagen piece to mark the location of the sample. Then, add a small amount of OCT to just cover the collagen. Let the block sit a few minutes to allow the OCT to infiltrate into the collagen. Then, put it on dry ice. Once the OCT just starts turning white, add more OCT to the top of the mold and wait until the entire block solidifies. Store at −80°C until prepared to start sectioning.

Samples were prepared for immunohistochemistry by making 6μm cryosections. Samples were stored at −80°C until prepared for immunohistochemistry.

### Fixed cell imaging of MIN6 cells and human cadaveric pancreatic islets

MIN6 cells were prepared for immunofluorescence according to standard lab protocols.^13^ In brief, cells were previously plated onto glass coverslips (12CIR-1.5 (Fisherbrand; Cat #: 12-545-81), after being passed through a cell strainer (Fisher Scientific; Cat #: 22-363-548) to ensure plating of single cells and to reduce cell clumping. This allows for better resolution of subcellular compartments via microscopy. After growing the cells and performing the different experimental conditions, cells were fixed at 37°C with 4% PFA/PBS (diluted from 16% stock; Electron Microscopy Sciences; Cat #: 100503-917) for 15 minutes. Coverslips were washed with PBS 4x before being permeabilized for 10 minutes with 0.1% TritonX-100 (Fisher Scientific; Cat #: AC21568-2500). Samples were incubated in block buffer (1% BSA/PBS) (Sigma; Cat #: A8806) for 1hr at room temperature and then incubated with primary antibody diluted in blocking buffer overnight at 4°C (see above for specific dilutions for different primary antibodies). Samples were then washed 4x with PBS and incubated with secondary antibody diluted 1:500 in blocking buffer for 1hr at room temperature in the dark. Coverslips were washed 2x with PBS and then incubated in DAPI (4′,6-Diamidino-2-phenylindole (DAPI), 10 mg/mL: (Biotium; catalog no. 40043)) diluted in PBS for 5 minutes at room temperature in the dark. Samples were then washed 2x with PBS before being mounted onto slides (VistaVision Microscope Slides, Histobond (75 mm by 25 mm by 1mm): (VWR; catalog no. 16005-110)) with SlowFade Gold Antifade reagent: (Invitrogen; Cat #: S36936).

For immunohistochemistry of human cadaveric islet cryosections, frozen slides were thawed for at least 30 minutes at room temperature before use. Samples were then prepared for immunofluorescence using a similar protocol as above, except that samples were incubated in secondary antibody for 2hrs rather than 1hr.

### Live cell imaging of human cadaveric pancreatic islets

Pancreatic human islets were imaged via time-lapse imaging using Stanford core facility’s Inverted Zeiss LSM 880 Laser Scanning Confocal Microscope at 40x magnification using controlled environmental conditions (37°C, 5% CO_2_). Images were collected every 5 minutes, along with z-stacks encompassing the entire islet. For each condition, we imaged each islet for at least 1hr. Perfect focus and Piezo stage movement capability enabled us to maintain the focal plane throughout the imaging window. Imaging acquisition conditions were maintained constant between all test groups to allow for ready comparison between samples (*e.g.*, gain, laser power, offset, etc.) FIJI imaging software was used to process the videos, and the same brightness, contrast, background subtraction, z-projection, cropping, and other processing settings were used across all samples to ensure accuracy of comparisons.

Islets were maintained in RPMI low glucose medium (no FBS) and supplemented with HEPES and beta-mercaptoethanol to maintain cell viability during the imaging window. Islets were placed in ibidi 8-well glass-bottom chambers (ibidi; Cat #: 80827-90) immediately before imaging. Cells were stained with the following live cell imaging dyes: SiR-Tubulin (100nM) for microtubule cytoskeleton (Cytoskeleton Inc.; Cat #: CY-SC002), BODIPY FL C5-Ceramide (5μM) for Golgi and plasma membrane (Thermofisher; Cat #: D3521), and Mitotracker Red CMXRos (200nM) for mitochondria (Thermofisher; Cat #: M7512). BODIPY and MitoTracker dyes were incubated with the islets for 30 minutes before washout, while SiR-Tubulin dye was maintained on the cells throughout the imaging experiment. The following treatment conditions were studied: low glucose (2.8mM), high glucose (16.7mM), high glucose + Exendin4 (20nM), high glucose + TUG891(50μM), high glucose + Exendin4 +TUG891. For HDAC6 inhibitor experiments, all the above conditions were also tested with a preincubation with HDAC6 inhibitor Ricolinostat (5nM) for 15minutes before inducing other treatments.

### Western Blot:

Post-treatment (5×10^6 cells) samples were collected and lysed in RIPA buffer, and clarified lysates were treated with an SDS loading buffer containing β-mercaptoethanol. Upon electrophoretic separation, Western blots were prepared using the eBlot L1 (Genscript) following the manufacturer’s protocols onto 0.2μm PVDF membranes (Thermo Fischer, Cat #: 22860). Post-transfer, membranes were blocked in Li-Cor Intercept^®^ (TBS) Blocking Buffer (Cat #: 927-60001) overnight at 4°C and cut with razor blade at appropriate molecular weight markers. Appropriate membrane slices were incubated with the following antibodies diluted in blocking buffer for 1hr at room temperature: (Anti-mouse Acetylated Tubulin 1:2000; Cat#: sc-23950), (Anti-Rabbit phosphor-pERK 1:1000; Cat #: 9107S), (Anti-Rabbit Lamin A+C 1:1000, Cell Signaling Cat#: 2032), (Anti-Rabbit Beta-Actin; 1:1000; Cat #: A1978). Membranes were washed 5x in TBST and incubated with Li-Cor secondary antibodies diluted in blocking buffer (Donkey anti-Rabbit IRDye 800 1:15000 and Donkey anti-mouse IRDye 680 1:15000). After 30 minutes of incubation at room temperature, membranes were washed in TBST and imaged on the LICOR Odyssey Imager.

### Image Processing/Quantification:

Image analysis was performed using FIJI imaging software, using different strategies based on the assay. For each experiment, all test groups were treated the same for image processing: z-stacking, subtracting background (along with Math-Subtract function after measuring background), adjusting brightness/contrast to baseline, cropping, etc. Representative images with scale bars are included for each experiment.

Ciliary length was assessed via manual measurements using the FIJI measuring tool.

The average volume of an individual mitochondrion for each condition was assessed using FIJI plugin 3D Mitochondrial Analyzer.^73^

Volume of microtubule density in the cell was measured by thresholding and creating a mask for the total cell area versus thresholding and creating a mask for the microtubule network. Default settings were used for thresholding, with manual inspection for each image to ensure that the mask corresponded well with the image. The area of each was measured, and the results were reported as the percentage of the total cell area that contained microtubules. Note that this is a measure of total microtubule density, not taking into account other features of microtubules like length, polymerized versus depolymerized pools, etc. A similar strategy was used for measuring acetylated microtubule density except that we measured the total percentage of microtubules that are acetylated (aka, =area of acetylated microtubules/area of total microtubules).

Cytoplasmic distribution of β-COP and HDAC6 was measured, after processing all images using the same parameters to subtract background, by generating ROIs for the cytoplasm of each cell and measuring the Mean Gray Value.

For all other quantification, yes-no binning was used to calculate the percentage of cells with the given phenotype, ~100 cells per replicate.

### Quantification and Statistical Analysis:

Data analysis and plotting was performed using Microsoft Excel and GraphPad Prism. One-Way ANOVA was used for statistical analysis, with p values = *P = 0.05; **P = 0.01; ***P=0.001; ****P = 0.0001. All experiments were performed in biological replicate. All image quantification was performed via FIJI imaging software. Final figures are prepared in BioRender.

## Supplementary Material

Supplement 1 **Figure S1: Western blot of pERK1/2 validates findings made via mass spectrometry.** Western blot for pERK1/2 (T202, Y204) phosphorylation confirming findings from mass spectrometry, with samples collected at 5min versus 20min post-stimulation. β-actin and Lamin are used here as loading controls.
**Figure S2: Workflow for identification of key kinase regulators of GSIS and their substrates**. Schematic of the kinase inhibitor screen workflow for identifying novel candidate kinases regulating GSIS. MIN6–6 cells (which secrete luciferase-tagged insulin^67^) are seeded, serum-starved for 24hr to induce cell synchrony, and incubated in low glucose along with the Grey Lab Inhibitor Library, which consists of 240 different inhibitors at 50μM each. Cells were then switched to high glucose+GLP1-R+FFAR4 agonist treatment for 30 minutes. Samples were then assessed via NanoGlo Luciferase plate-reader assay before being binned based on the degree with which each kinase inhibitor increases/decreases GSIS compared to baseline. We can then focus on the functions of a specific kinase of interest by assessing phosphosite abundance in High Glucose + TUG891 + Exendin-4 treatment with and without supplementation with inhibitor treatment. All sites were scored based on kinome motifs and ranked based on fold change/p-value. The changes in site abundance sorted based on kinase motif was used as a tool for predicting the effects of different inhibitor treatments on different kinase activities.
**Figure S3: Plot showing the dose-response effects of HDAC6i treatment on differential GSIS treatments in GSIS.** Cells were preincubated with different doses of HDAC6i (with DMSO as control) for 15 min before stimulation with the following GSIS conditions: LG, HG, HG+TUG891, HG+Exendin4, and HG+TUG891+Exendin4 on GSIS. The following doses of HDAC6i were tested: 5nM, 50nM (IC_50_), 500nM. N=3
**Figure S4: Representative image showing ATAT1 co-localization with cis Golgi (GM130) in MIN6 cells.** Images were collected at 63x magnification; green= ATAT1, red= GM130, white= acetylated tubulin, blue= DAPI. Scale = 10μm.
**Figure S5: ATAT1 has differential localization to the nucleus upon stimulation with different GSIS conditions.** Z-stack immunofluorescent images collected via confocal microscopy at 63x magnification at 5min versus 20min post-stimulation. Images show the following markers for localization: ATAT1 (green) and DAPI (blue). Scale bar= 10μm.
**Figure S6: ATAT1 has differential cytoplasmic distribution upon stimulation with different GSIS conditions.** Z-stack immunofluorescent images collected via confocal microscopy at 63x magnification at 5 versus 20min post-stimulation. The following markers were used for staining: ATAT1 (green), -tubulin (red), acetylated tubulin (white), and DAPI (blue). Scale bar= 10μm. This is an expanded version of Figure 6H.
**Figure S7: GSIS of healthy adult human cadaveric islet using perifusion assay to measure insulin secretion.** This is the same batch of human islets used for performing phosphoproteomics. Concentration of insulin (mU/L) was measured at 15minutes, the same time point at which the human phosphoproteomics was performed. Error bars = SEM. Patient was non-diabetic, BMI=37, female, 18yo.
**Figure S8: Live cell imaging of differential GSIS conditions in cadaveric human islet reveals conserved context-specific organellar dynamics.** Healthy human cadaveric islets from 3 different donors of similar ages, BMI, and health status were imaged via time-lapse confocal microscopy at 40x magnification (oil). Z-stack images were collected every 5 minutes to enable resolution of the following features: microtubules (SiR Tubulin far red dye), Golgi (ceramide dye), and mitochondria (CMX-ROS). Scale bars = 10m. Images shown here were collected across the full time course (as opposed to the 15min time point shown in Fig. 7B, which was selected because of its correspondence to the time point chosen for mass spectrometry). Note that these images are insets of only a few cells from a given islet to enable better resolution of intracellular compartments.
