# Supplementary figures and images for: Metabolic STAMP for deciphering GPCR-regulated insulin secretion by pancreatic β cells
Source: bioRxiv. 2025 Oct 4:2025.10.03.680349. Preprint. [Version 1] doi: 10.1101/2025.10.03.680349 (PMC12621840; doi:10.1101/2025.10.03.680349)

**FIG.S1**

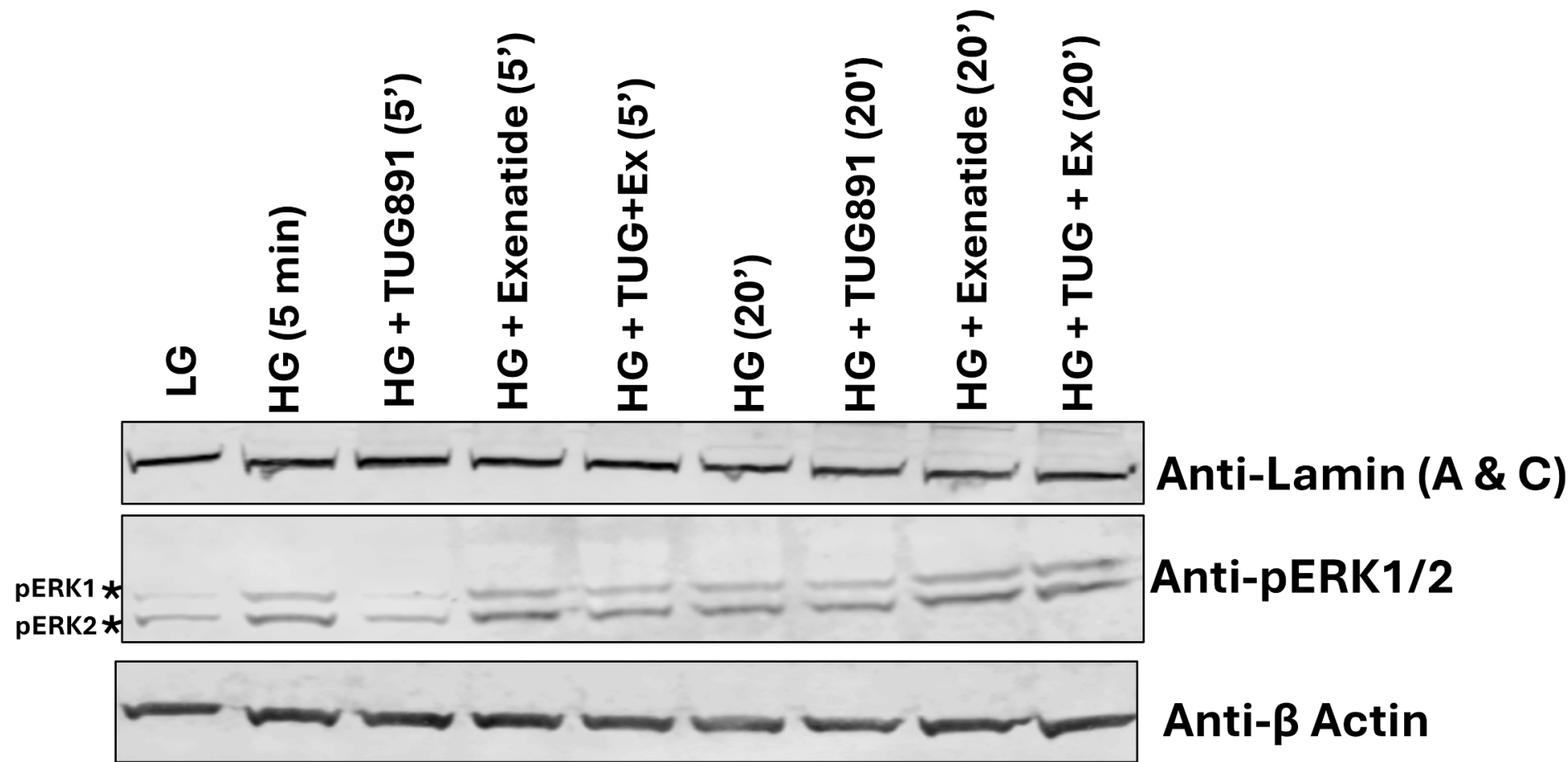

FIG.S2

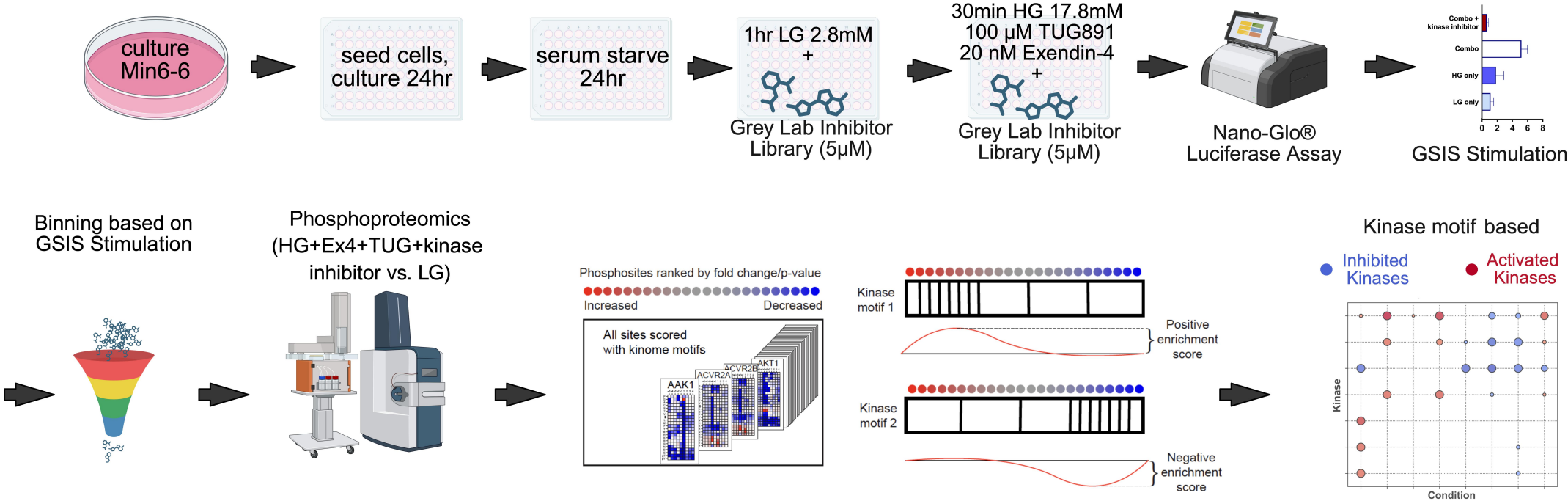

FIG.S3

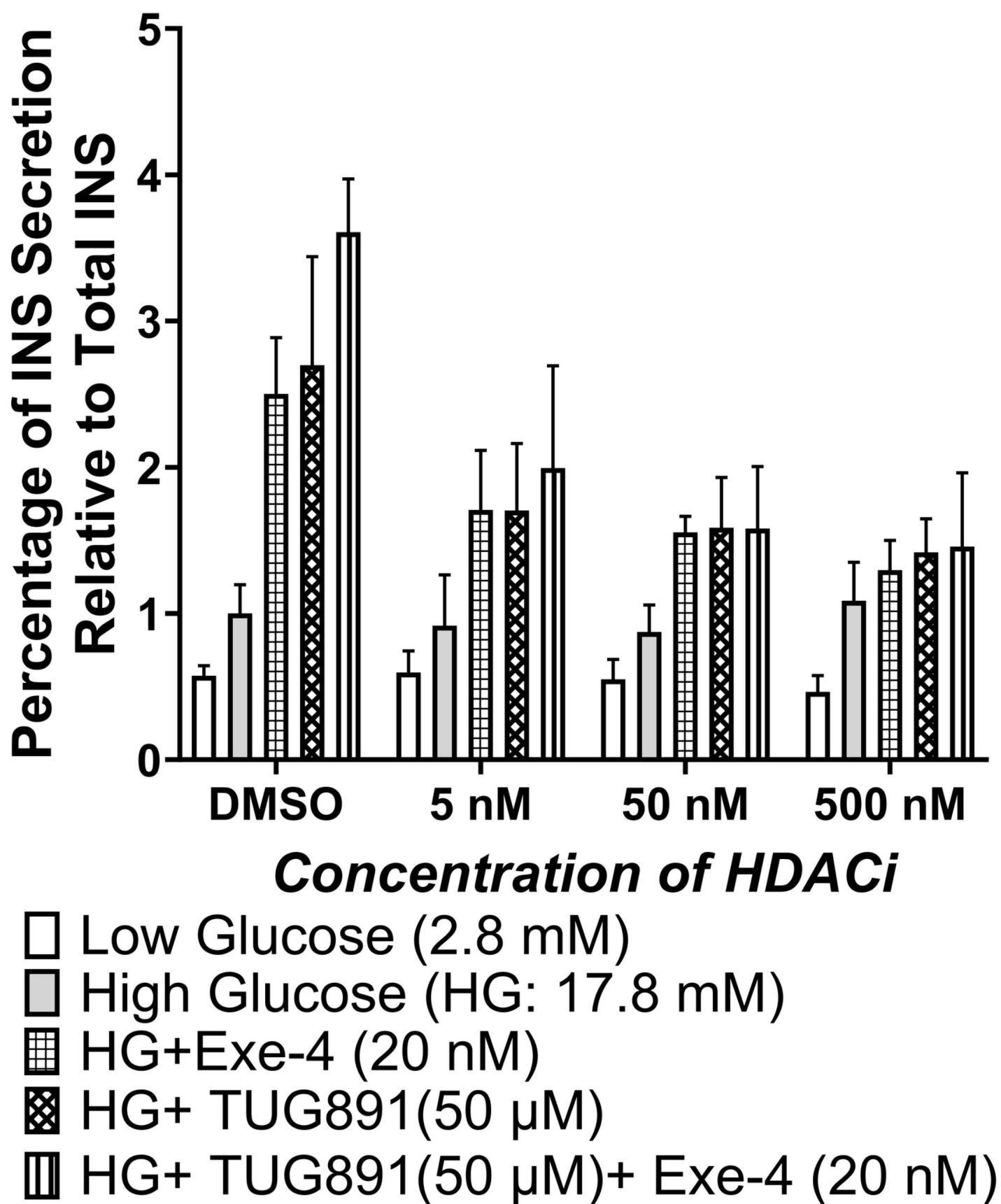

FIG.S4

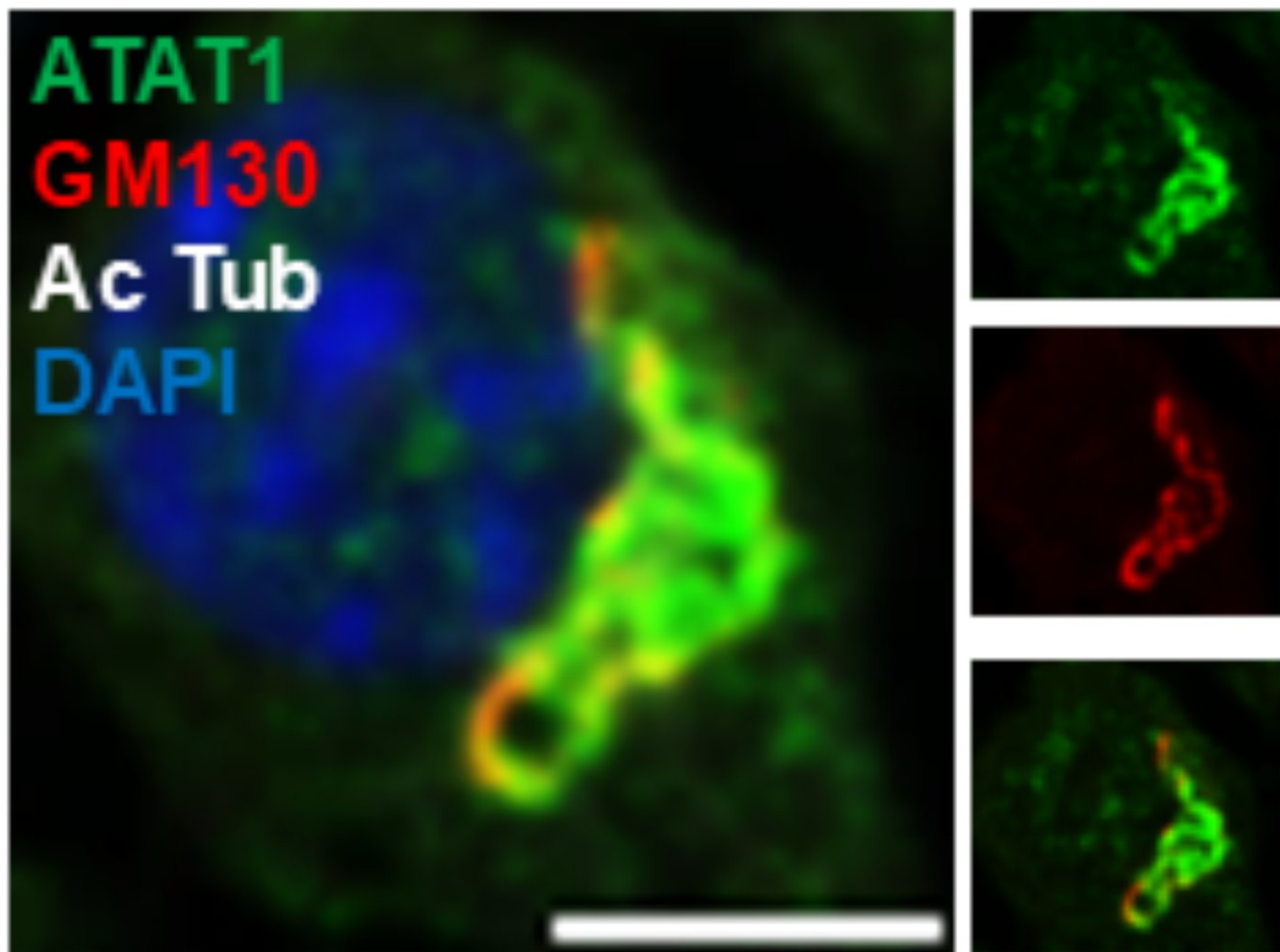

FIG.S5

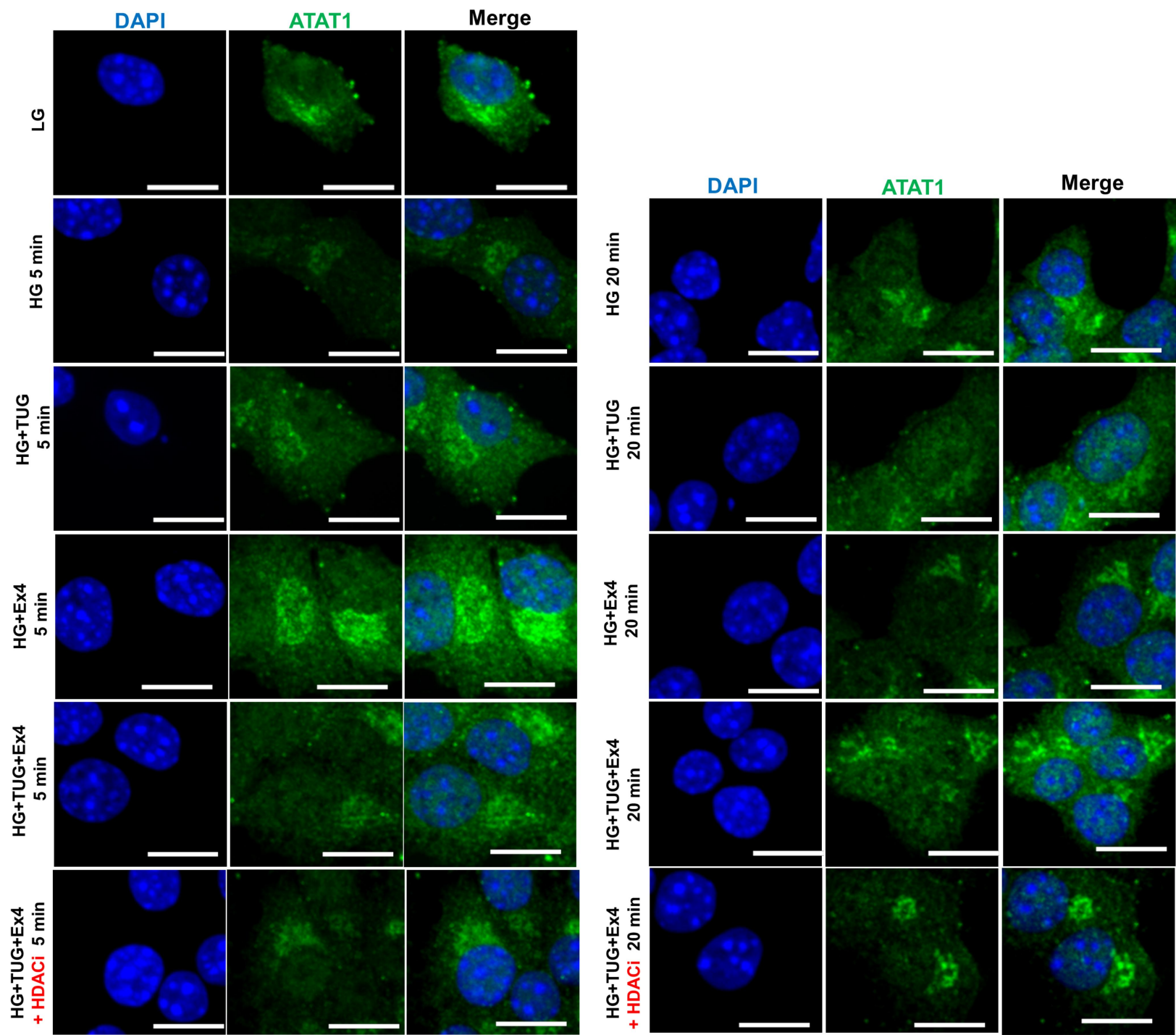

FIG.S6

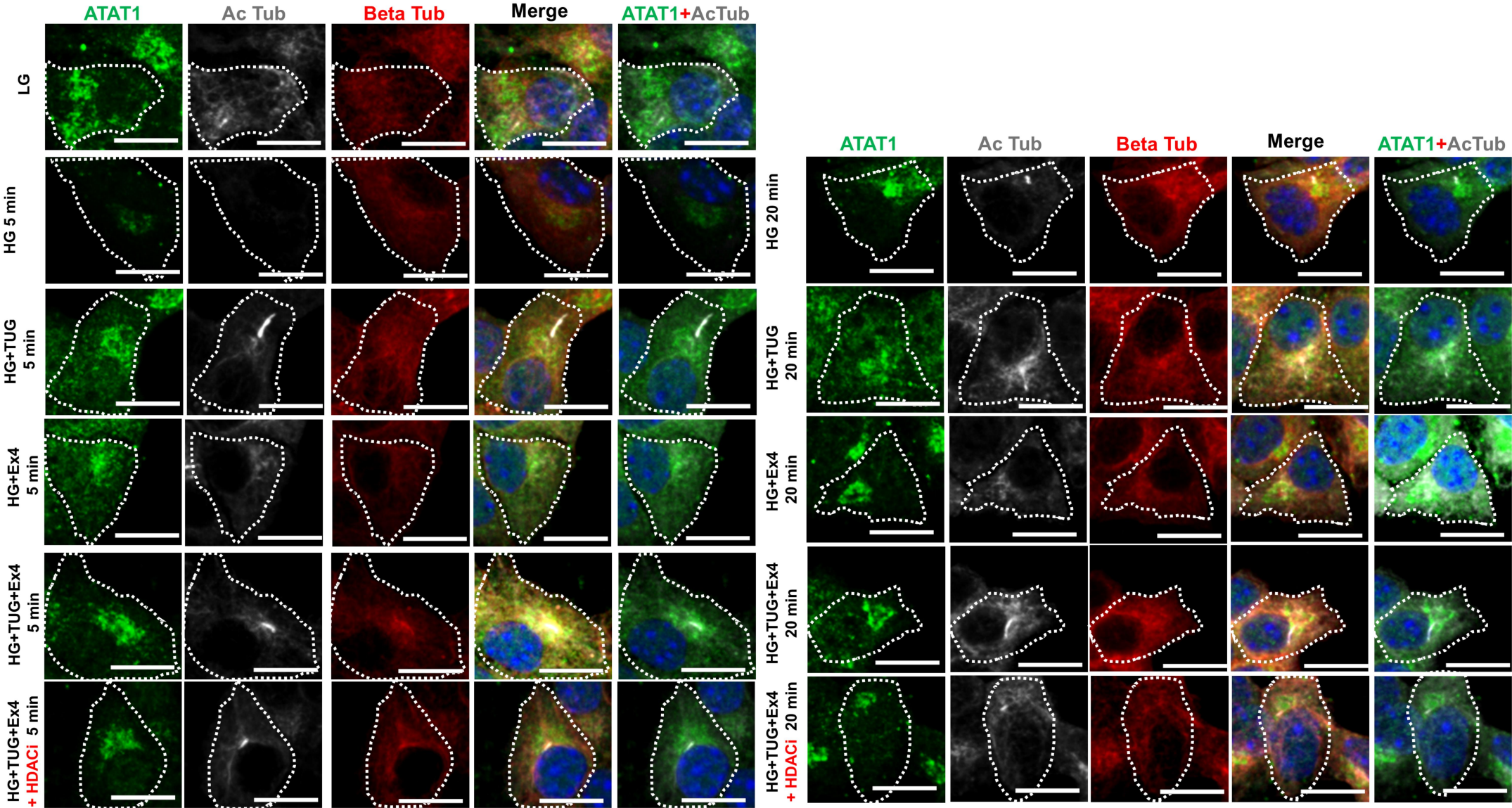

FIG.S7

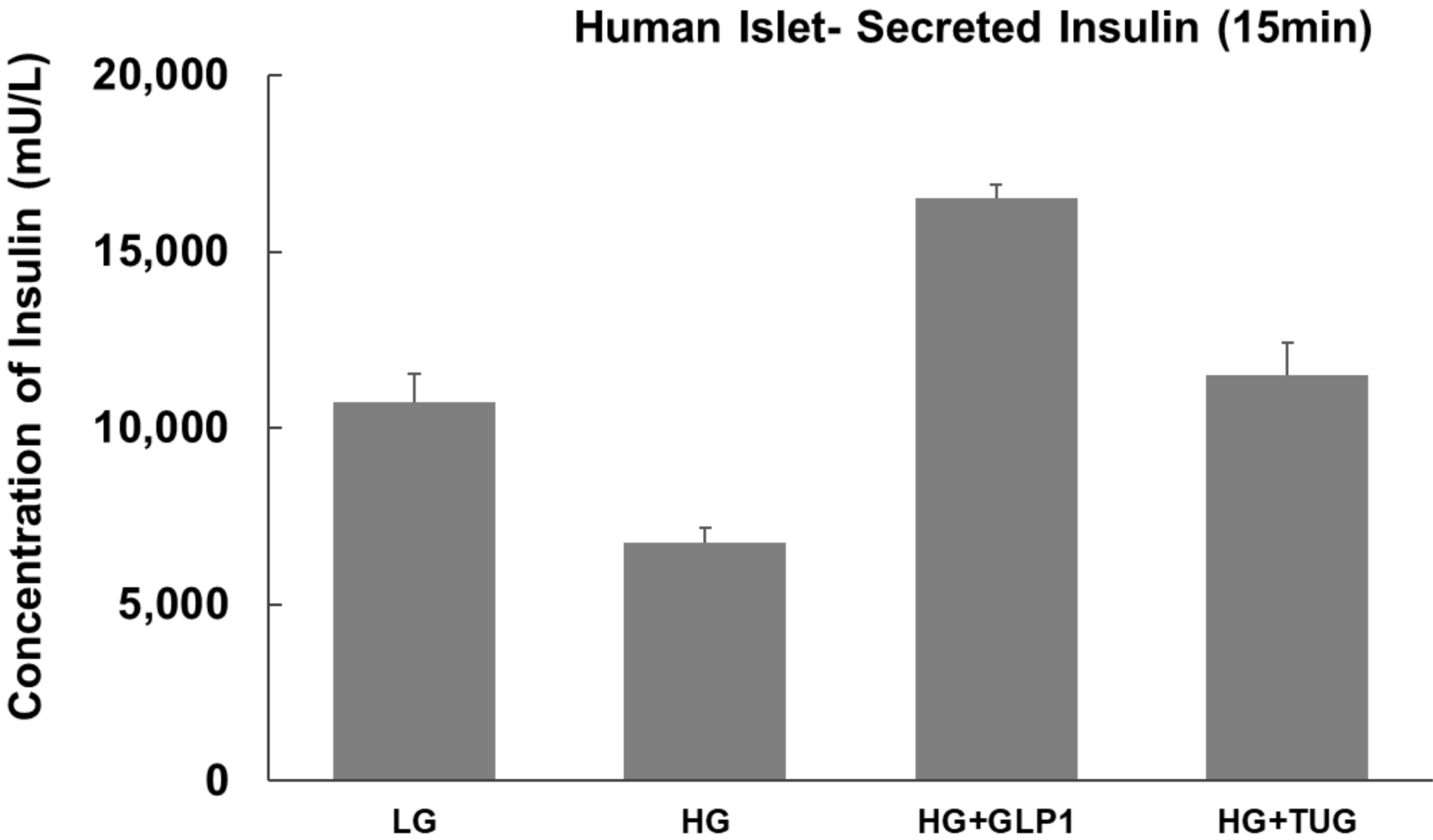

FIG.S8

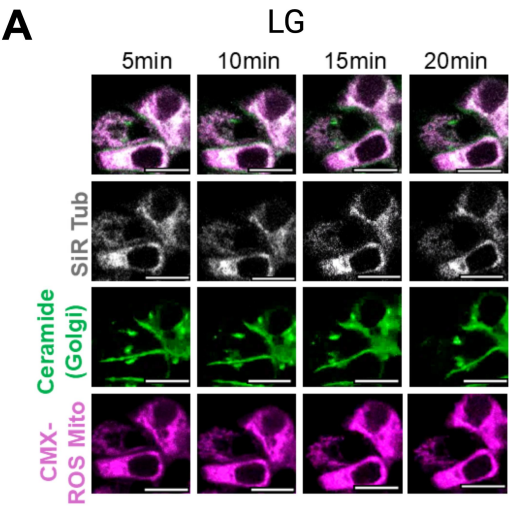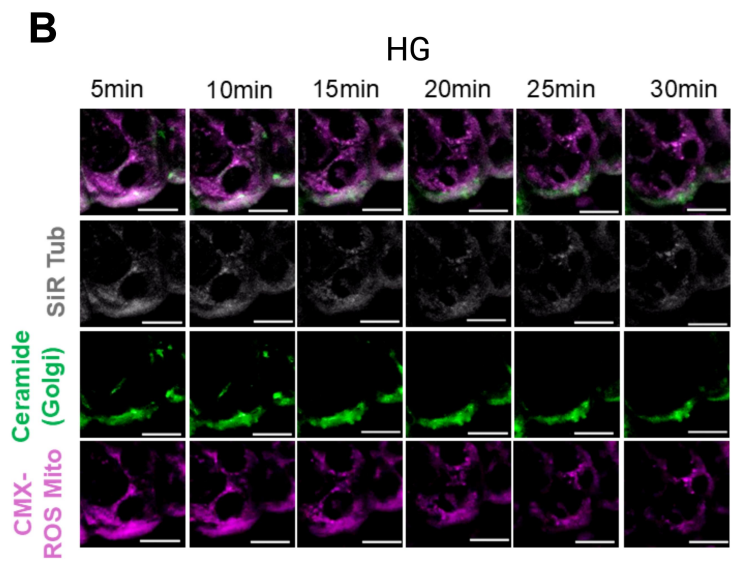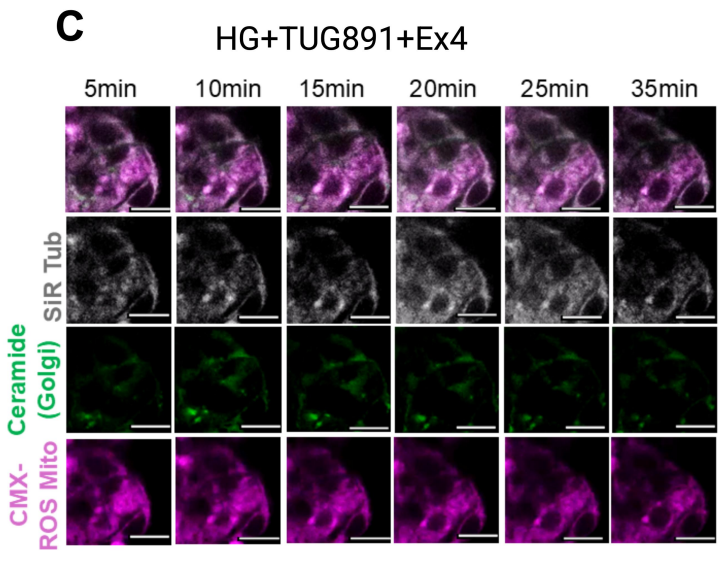

Supplement: Supplement 1 — Figure S1: Western blot of pERK1/2 validates findings made via mass spectrometry. Western blot for pERK1/2 (T202, Y204) phosphorylation confirming findings from mass spectrometry, with samples collected at 5min versus 20min post-stimulation. β-actin and Lamin are used here as loading controls. Figure S2: Workflow for identification of key kinase regulators of GSIS and their substrates. Schematic of the kinase inhibitor screen workflow for identifying novel candidate kinases regulating GSIS. MIN6–6 cells (which secrete luciferase-tagged insulin67) are seeded, serum-starved for 24hr to induce cell synchrony, and incubated in low glucose along with the Grey Lab Inhibitor Library, which consists of 240 different inhibitors at 50μM each. Cells were then switched to high glucose+GLP1-R+FFAR4 agonist treatment for 30 minutes. Samples were then assessed via NanoGlo Luciferase plate-reader assay before being binned based on the degree with which each kinase inhibitor increases/decreases GSIS compared to baseline. We can then focus on the functions of a specific kinase of interest by assessing phosphosite abundance in High Glucose + TUG891 + Exendin-4 treatment with and without supplementation with inhibitor treatment. All sites were scored based on kinome motifs and ranked based on fold change/p-value. The changes in site abundance sorted based on kinase motif was used as a tool for predicting the effects of different inhibitor treatments on different kinase activities. Figure S3: Plot showing the dose-response effects of HDAC6i treatment on differential GSIS treatments in GSIS. Cells were preincubated with different doses of HDAC6i (with DMSO as control) for 15 min before stimulation with the following GSIS conditions: LG, HG, HG+TUG891, HG+Exendin4, and HG+TUG891+Exendin4 on GSIS. The following doses of HDAC6i were tested: 5nM, 50nM (IC50), 500nM. N=3 Figure S4: Representative image showing ATAT1 co-localization with cis Golgi (GM130) in MIN6 cells. Images were collected a [file media-1.pdf]
